# Rice Genotype-Dependent Phyllosphere Microbiome Assembly and Isolation of Antagonistic *Burkholderia* for Sheath Blight Biocontrol

**DOI:** 10.3390/ijms27114879

**Published:** 2026-05-28

**Authors:** Andrews Danso Ofori, Zohreh Nasimi, Muhammad Irfan Ahmed, Yaoting Yan, Wang Li, Abdul Ghani Kandro, Kazunori Okada, Keiichi Mochida, Yoshiteru Noutoshi, Aiping Zheng

**Affiliations:** 1 State Key Laboratory of Crop Gene Exploration and Utilization in Southwest China, Sichuan Agricultural University, Chengdu 611130, China; andrewsdanso@icloud.com (A.D.O.); z.nasimi@sicau.edu.cn (Z.N.);; 2 Department of Plant Pathology, Rice Research Institute, Sichuan Agricultural University, Chengdu 611130, China; 3 Agro-Biotechnology Research Center, The University of Tokyo, Bunkyo-ku 113-8657, Tokyo, Japan; 4 Bioproductivity Informatics Research Team, RIKEN Center for Sustainable Resource Science, Yokohama 230-0045, Kanagawa, Japan; 5 Microalgae Production Control Technology Laboratory, RIKEN Baton Zone Program, RIKEN Cluster for Science, Technology and Innovation Hub, Saitama 351-0198, Japan; 6 Kihara Institute for Biological Research, Yokohama City University, Yokohama 244-0813, Kanagawa, Japan; 7 School of Information and Data Sciences, Nagasaki University, Nagasaki 852-8521, Japan; 8 Graduate School of Environmental and Life Science, Okayama University, Okayama 700-8530, Japan; noutoshi@okayama-u.ac.jp

**Keywords:** rice phyllosphere, *Rhizoctonia solani*, sheath blight, *Burkholderia vietnamiensis*, biocontrol, microbiome, 16S rRNA sequencing, plant growth-promoting bacteria, disease resistance

## Abstract

Rice sheath blight (RSB), caused by *Rhizoctonia solani*, causes 10–50% yield losses globally. Using 16S rRNA sequencing of 100 rice cultivars, we found that resistant varieties harbor significantly more diverse bacterial communities (3230 OTUs; 2064 unique) than susceptible cultivars (599 OTUs; 36 unique). Resistant varieties were enriched in beneficial *Burkholderiaceae*, *Bacillaceae*, and *Pseudomonadaceae*, while *Sphingobacteriaceae* and *Enterobacteriaceae* were predominant in the susceptible rice varieties. From the 260 bacterial isolates, *Burkholderia vietnamiensis* J14EPLEAF2 presented potent antifungal activity (77% inhibition), suppressed lesion development, and abolished sclerotia formation. This strain displayed multiple plant growth-promoting traits, enhanced seed germination, and primed defense responses by upregulating PR5 and PR10. Hypersensitive response assays confirmed *B. vietnamiensis* as non-pathogenic, unlike *B. gladioli* and *B. cepacia*. This study identifies *B. vietnamiensis* J14EPLEAF2 as a promising, safe biocontrol agent for sustainable rice disease management.

## 1. Introduction

Rice (*Oryza sativa* L.) is the principal crop for more than half of the world’s population, accounting for over 23% of global caloric intake [1,2]. Numerous biotic constraints are threatening rice production. Among them, sheath blight, caused by the necrotrophic basidiomycete *Rhizoctonia solani* Kühn (anastomosis group AG-1 IA), has been regarded as one of the most dangerous diseases affecting rice worldwide [3,4]. The disease is reported to lead to yield losses of 10–30% and up to 50% under highly favourable disease-promoting conditions [5,6]. Whereas many fungal pathogens have restricted host and geographic ranges, the host range of *R. solani* AG-1 IA is very wide, having been demonstrated to be pathogenic to rice, soybean, maize, and several other important crops across 32 plant families [7,8].

Rice is an economically important crop worldwide, and no major resistance genes against *R. solani* have been identified. Some cultivars show partial quantitative resistance [9]. Chemical fungicides are used to manage the disease, but their use poses several environmental hazards and has led to the development of many resistant strains, including Jinggangmycin resistance reported in China [10]. There is a need to understand the molecular mechanisms underlying *R. solani*’s pathogenicity and to develop sustainable alternatives.

The phyllosphere, which comprises the plant’s aerial parts, is a vast ecosystem that hosts diverse microorganisms, including archaea, bacteria, fungi, protozoa, cyanobacteria, viruses, and nematodes [1]. Microbiota provide important functions in maintaining healthy plants through mechanisms such as nutrient acquisition, stress resistance, and support of plant growth [11]. The 16S rRNA gene amplicon sequencing approach has been widely used to explore the structure, composition, and spatiotemporal patterns of microbial communities on leaf surfaces (phyllosphere) [12]. Bacterial cell populations in the phyllosphere can range from 10^5^ to 10^7^ cells/cm^2^ of leaf area, with significant representation by three major phyla: Proteobacteria, Actinobacteria, and Bacteroidetes [13,14].

The plant genotype drives the plant’s phyllosphere microbiome; specific assemblies of microbes have been shown to suppress disease through many studies [15,16], and typically, these resistant genotypes will host unique communities enriched with beneficial taxa such as members of *Burkholderiaceae*, *Bacillaceae* and *Pseudomonadaceae*, whereas susceptible genotypes tend to host communities that are dominated by opportunistic or relatively weakly competitive bacterial taxa [16,17]. This pattern supports the notion that the microbiome is heritable and therefore potentially provides a basis for using resistance-associated microbiome signatures as breeding markers or biocontrol agents [18].

A key characteristic that makes *Burkholderia* particularly interesting compared to other phyllosphere-associated bacteria is its duality in both ecological position and metabolic versatility [19]. Some *Burkholderia* species can be opportunistic pathogens of both humans and plants (e.g., *B. cepacia* complex and *B. gladioli*), while other species are beneficial plant-associated symbionts that promote growth and reduce disease through antibiotics, resource competition, and ISR [20,21]. To evaluate whether a specific *Burkholderia* strain is suitable for biocontrol applications, it will be necessary to assess each strain with sufficient specificity, given its dual reputation.

In this study, we examined the phyllosphere microbiome of 100 rice cultivars with varying susceptibility to sheath blight to determine whether resistance is associated with a unique, potentially heritable microbiome signature. We then functionally characterized antagonistic *Burkholderia* strains from resistant cultivars and evaluated their biocontrol efficacy, biosafety, and potential to promote plant growth.

## 2. Results

### 2.1. Phyllosphere Microbiome Composition Differs Between Resistant and Susceptible Cultivars

**Figure 1:**
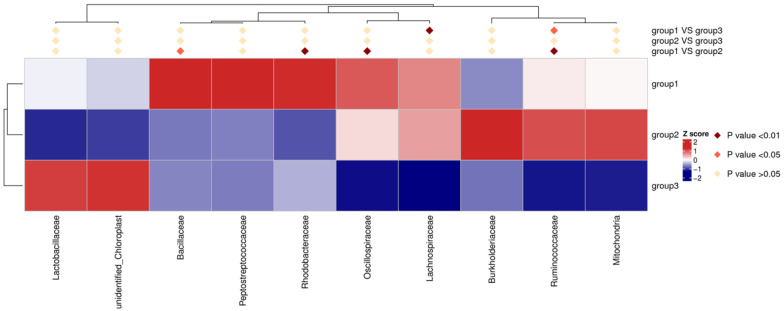
Metastat analysis of beta diversity among groups at the family level. A heat map of the differential abundance of bacterial families for all pairwise comparisons of rice resistance groups (group1 vs. group2, group2 vs. group3, and group1 vs. group3). The z-scale in the legend represents the relative abundance of bacteria at the family level; red indicates the highest abundance, white indicates an equal distribution of samples, and blue indicates either a lack of or less abundant members. Differences in microbial community structure were found among resistant (group 1), moderately resistant (group 2), and susceptible (group 3) rice varieties, as supported by PERMANOVA (*p* < 0.001).

The endophytic bacterial communities in the phyllosphere of 100 rice cultivars with varying susceptibility to *R. solani* were profiled using 16S rRNA amplicon sequencing. *Cyanobacteria*, *Proteobacteria*, *Firmicutes*, *Bacteroidota*, and *Actinobacteriota* were the most prevalent phyla across all resistance groups, spanning 34 bacterial phyla (Supplementary File S1). Nevertheless, the community structure of resistant (group 1), moderately resistant (group 2), and susceptible (group 3) cultivars exhibited substantial differences (Figure 1; PERMANOVA, *p* < 0.001).

The most significant phyla found to be highly abundant in resistant and moderately resistant rice varieties included *Actinobacteriota*, *Myxococcota*, *Chloroflexi*, *Halobacterota*, *Gemmatimonadota*, *Nitrospirota*, *Planctomycetota*, and *Proteobacteria*. In contrast, the susceptible rice varieties exhibited higher levels of abundance of *Cyanobacteria*, *Deferribacterota* and *Deinococcota* compared to the non-susceptible rice varieties. A summary representation of the abundance of each phylum at the group level across all three resistance categories is shown in Supplementary Figure S1.

**Figure 2:**
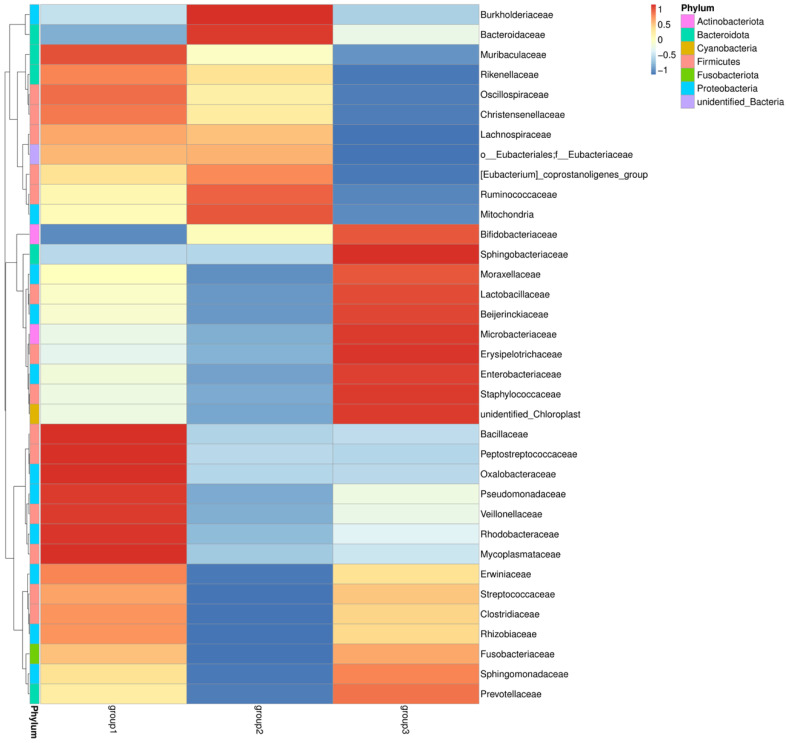
Taxon abundance heatmap of bacterial communities in the rice phyllosphere at the family level. Heatmap displaying the relative abundance of bacterial families across three rice resistance groups: group 1 (highly resistant varieties), group 2 (mid-tolerant varieties), and group 3 (susceptible varieties). Values are scaled by taxon relative abundance, with blue indicating lower relative abundance or absence, and red indicating higher taxon concentration in the samples. The cluster dendrogram at the top represents hierarchical clustering of samples based on community similarity, with branch colors corresponding to bacterial phyla. Resistant and tolerant varieties (groups 1 and 2) show enrichment of families including *Burkholderiaceae*, *Bacillaceae*, *Pseudomonadaceae*, and *Oxalobacteraceae*, while *Sphingobacteriaceae*, *Enterobacteriaceae*, and *Staphylococcaceae* dominate susceptible varieties (group 3).

At the family level, *Burkholderiaceae*, *Bacillaceae*, *Pseudomonadaceae*, *Oxalobacteraceae*, *Bacteroidaceae*, and *Rhodobacteraceae* were the most prevalent in the phyllosphere microbial communities of resistant and tolerant rice varieties as compared to *Sphingobacteriaceae*, *Moraxellaceae*, *Enterobacteriaceae*, and *Staphylococcaceae* that dominated susceptible rice types (Figure 2).

**Figure 3:**
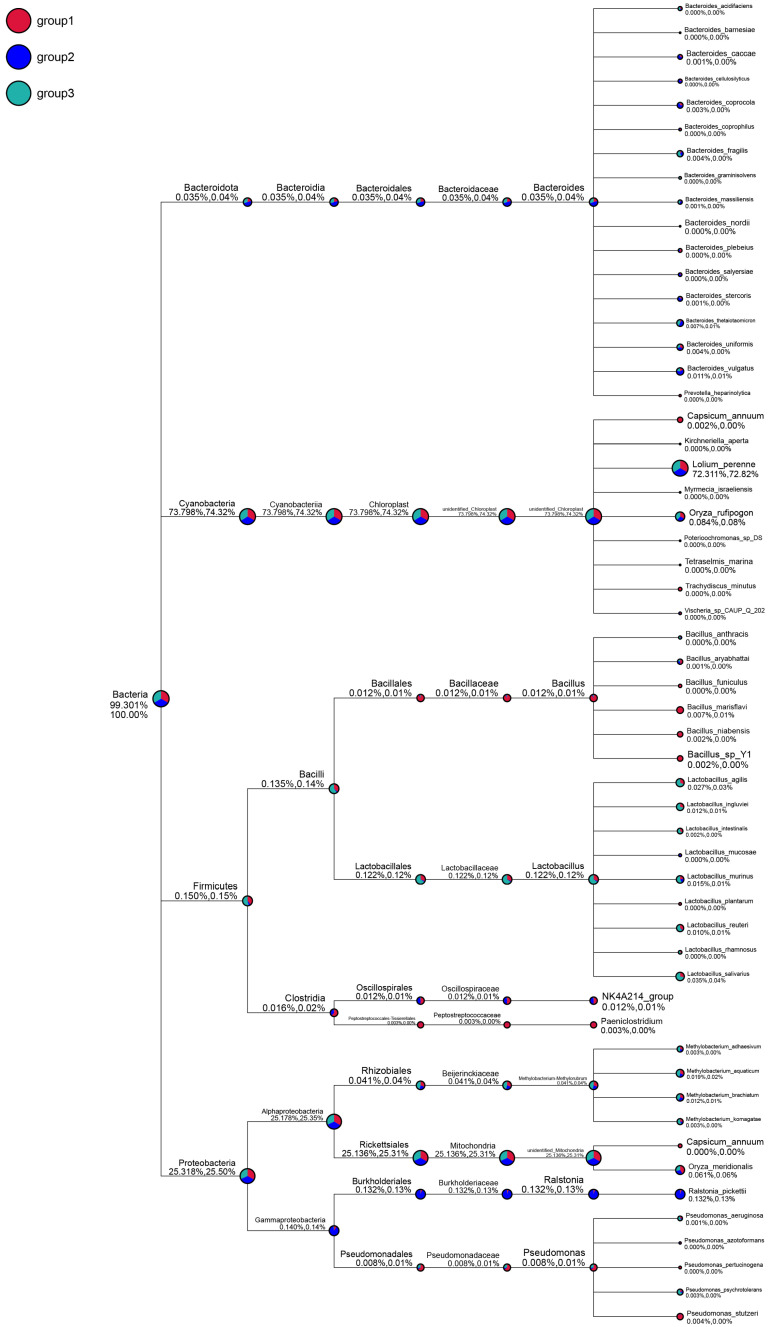
Taxonomy composition and abundance of the rice phyllosphere. The larger area of the colored pie chart at a branch represents the greater bacterial abundance of the taxon in the group. Red: highly resistant rice varieties, blue: tolerant rice varieties, green: susceptible rice varieties.

At the genus level, *Bacillus*, *Pseudomonas*, *Paeniclostridium*, and *Ralstonia* were relatively more abundant in resistant and moderately resistant varieties, whereas *Lactobacillus* and *Methylobacterium* were more prevalent in susceptible cultivars (Figure 3; Supplementary Figure S2).

### 2.2. Resistant Cultivars Harbor Higher Microbial Diversity and Unique OTU Reservoirs

**Figure 4:**
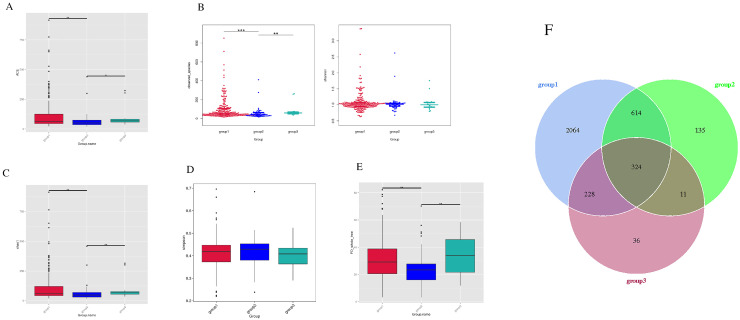
Alpha Diversity analysis of microbial community for each group of rice varieties. Group 1: highly resistant rice varieties, group 2: tolerant rice varieties, group 3: susceptible rice varieties. (**A**) ACE index showing significant differences among groups (* *p* = 0.01, ** *p* = 5 × 10^−7^). (**B**) Observed species richness and Shannon diversity index indicating significant variation (*** *p* < 2 × 10^−4^, ** *p* < 0.0043). (**C**) Chao1 richness estimator (** *p* < 0.008). (**D**) Simpson diversity index. (**E**) Phylogenetic diversity (PD whole tree) showing significant differences among groups (** *p* < 0.007). (**F**) Venn diagram illustrating the distribution of unique and shared operational taxonomic units (OTUs) among the three groups. Resistant varieties (Group 1) harbor the highest number of unique OTUs, followed by tolerant (Group 2) and susceptible (Group 3) varieties, indicating that resistant genotypes support more diverse and complex microbial communities.

Phyllosphere microbial community richness differed between rice resistance groups, as evaluated using alpha-diversity analysis (Table 1; Figure 4). Resistant genotypes had significantly higher microbial richness than tolerant and susceptible genotypes. Richness-based observed species richness was highest in the resistant group (94 OTUs), compared to 55 OTUs in the tolerant group and 78 OTUs in the susceptible group (Table 2). This effect was also reflected in Chao1 estimates of total richness, which showed significantly higher values in resistant genotypes (109.571) compared to tolerant genotypes (64.074).

Venn diagram analysis revealed highly disparate OTU numbers and compositions across groups. Resistant rice varieties harbored 3230 total OTUs, of which 2064 were unique to resistant varieties. An alternative visualization of the OTU distribution patterns among resistance groups is presented in Supplementary Figure S3. In contrast, tolerant varieties had 1084 OTUs, including 135 unique OTUs; susceptible varieties had only 599 OTUs total, with only 36 unique to susceptible varieties (Figure 4F). The high number of unique OTUs in resistant varieties is consistent with the idea that resistant genotypes preferentially recruit a more diverse and complex microbial community.

### 2.3. Beta Diversity Separates Communities by Resistance Class

**Figure 5:**
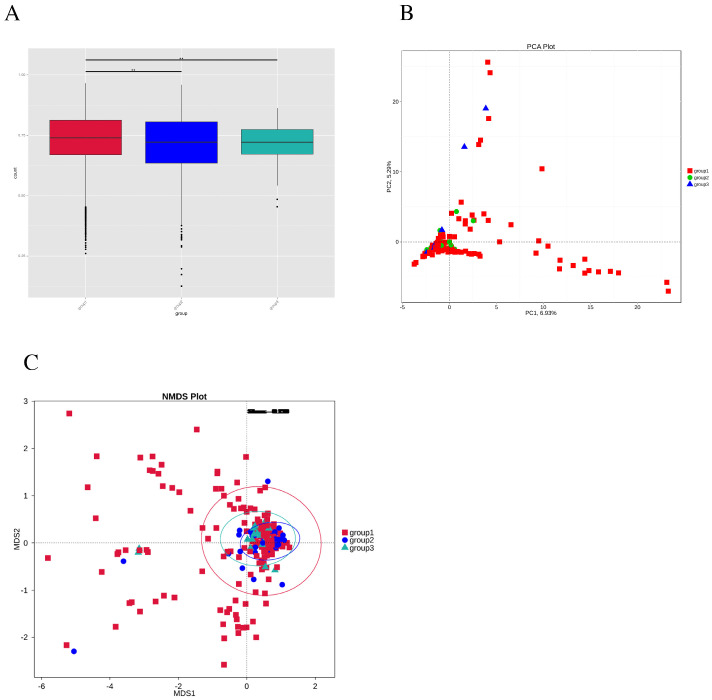
Beta-diversity of bacterial communities in the phyllosphere of rice plants differing in their level of resistance. (**A**) Histogram for beta-diversity based on the un-weighted UniFrac distances; Wilcoxon-test: group 1 vs. group 2 (*p* ≤ 0 **); group 1 vs. group 3 (*p* ≤ 0.007 **); group 2 vs. group 3 (no significance). (**B**) PCA showed that microbial communities clustered by plant resistance level. PC1 explained 6.93% of all variation in the community composition, and PC2 explained 5.29%. The clusters of microbial communities are quite similar in the high-resistance (group 1) and tolerant (group 2) rice varieties, whereas the susceptible (group 3) varieties clearly formed a separate cluster. (**C**) NMDS-ordination of the data based on unweighted UniFrac distances confirmed the clear separation of microbial communities across different levels of plant resistance. Group 1: high-resistance rice varieties; group 2: tolerant rice varieties; group 3: susceptible rice varieties.

Significant differences in microbial community composition were observed among rice resistance groups based on OTU relative abundance (Figure 5A). Pairwise comparisons revealed statistically significant differences between resistant and susceptible rice varieties, as well as between tolerant and susceptible varieties. In contrast, no significant difference was detected between resistant and tolerant groups (Supplementary Table S1), indicating that microbial community structure is most strongly differentiated between susceptible varieties and the two more resistant categories.

Principal component analysis (PCA) further revealed distinct clustering of microbial communities by resistance class (Figure 5B). Hierarchical UPGMA clustering based on community composition further supported the separation of resistance groups, with resistant and tolerant varieties clustering separately from susceptible varieties (Supplementary Figure S4). The first principal component accounted for 6.93% of the total variation, indicating that resistance status is a major driver of community differentiation. Samples from resistant and tolerant varieties largely overlapped, whereas susceptible varieties formed a more distinct cluster.

Unweighted UniFrac distance analysis revealed highly significant differences (*p* < 0.001; Figure 5C) in phylogenetic communities across all groups. Analysis using weighted UniFrac distances, which accounts for the relative abundance of taxa, confirmed these patterns and is presented in Supplementary Figure S5. This composition- and phylogeny-sensitive metric demonstrates that differences between categories are not confined to shifts in the abundance of specific taxa, but extend to evolutionary divergence of community members. Analysis of molecular variance (AMOVA) based on unweighted UniFrac (Table 3) distances revealed significant differences in microbial community composition among rice resistance groups (*p* = 0.01). Pairwise comparisons showed significant differentiation between resistant and susceptible varieties (*p* = 0.049), resistant and tolerant varieties (*p* = 0.043), and notably between tolerant and susceptible varieties (*p* = 0.015).

Pairwise and global comparisons of microbial community composition between rice resistance groups using unweighted UniFrac AMOVA. Values represent SS (Sum of Squares), df (degrees of freedom), MS (Mean Squares), and FS (Pseudo-F statistic). Numbers outside parentheses indicate the effect of the grouping factor, while numbers inside parentheses indicate the residuals. Group 1: high-resistance rice varieties; Group 2: tolerant rice varieties; and Group 3: susceptible rice varieties. Significant differences were detected between all pairwise comparisons: group1 vs. group3 (*p* ≤ 0.049), group1 vs. group2 (*p* ≤ 0.043), and group2 vs. group3 (*p* ≤ 0.015). Overall comparison across all three groups also showed significant differentiation (*p* ≤ 0.01). These results confirm that microbial community structure differs significantly across all resistance classes, with the greatest differentiation observed between tolerant and susceptible varieties.

**Figure 6:**
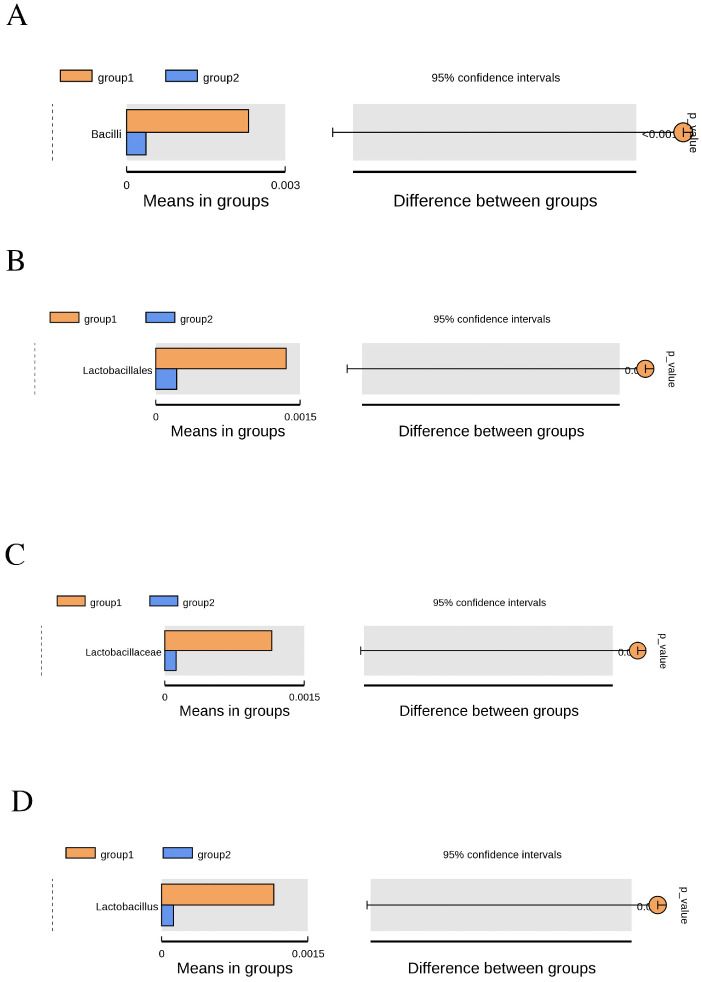
Bacterial taxa differential abundance analysis in multiple taxonomic classes between resistant and susceptible rice varieties. *t*-test bar plot comparing abundance of bacterial taxa of resistant (group 1) and susceptible (group 2) rice varieties at (**A**) class level, (**B**) order level, (**C**) family level, and (**D**) genus level (*p* < 0.001). Bar length represents the mean abundance for each group at that taxonomic classification; 95% confidence limits are shown on the horizontal bar. Right panel displays the differences in abundance for each taxon between the two rice variety groups, along with their respective confidence intervals.

At the genus level, *Lactobacillus* was found to be significantly (*p* < 0.001; Figure 6) more abundant in susceptible versus the resistant rice varieties. This may suggest that this taxonomic group plays a role in disease susceptibility. Further details are presented in Supplementary Figure S6 for the class, order, and family levels of the Metastats analysis.

### 2.4. Developmental Stage Influences Phyllosphere Community Dynamics

**Figure 7:**
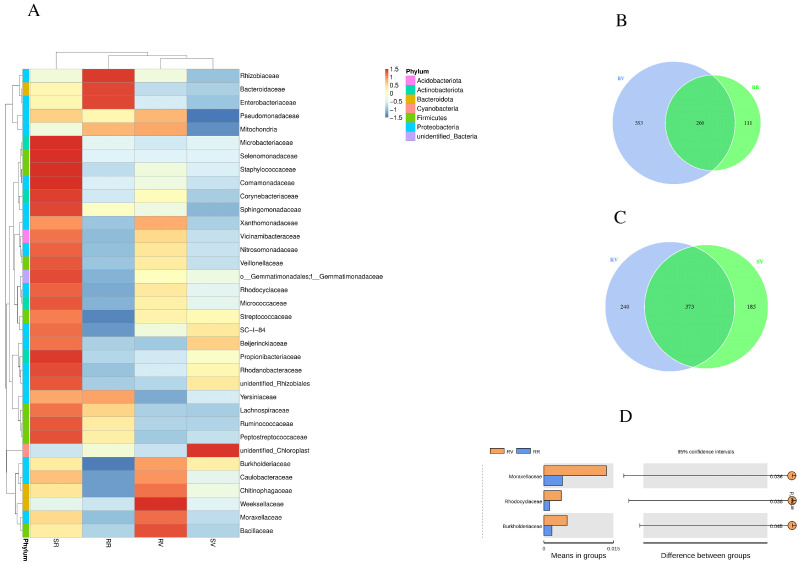
Comparison between microbial community of vegetative and reproductive stage of rice growth in susceptible and resistance rice varieties. (**A**) Taxa heatmap of related abundance at the family level. RV: vegetative growth of resistance varieties, RR: reproductive growth of resistance varieties, SV: vegetative growth of susceptible varieties, SR: reproductive growth of susceptible varieties. (**B**) Venn diagram of microbial communities with unique and shared OTUs among RV and RR. (**C**) Venn diagram of microbial communities with unique and shared OTUs among RV and SV. (**D**) Analysis of significantly differential microbiome between vegetative and reproductive of resistance varieties by *t*-test-bar-plot at *p* < 0.05.

Comparisons between vegetative and reproductive stages in resistant and susceptible varieties revealed clear temporal shifts in community composition and diversity (Figure 7). In resistant lines, *Burkholderiaceae*, *Bacillaceae*, *Chitinophagaceae*, and *Caulobacteraceae* were prominent in the vegetative stage, and *Burkholderiaceae*, *Rhodocyclaceae*, and *Moraxellaceae* showed higher abundance in vegetative leaves than in reproductive leaves (Figure 7A,D). Resistant varieties harbored numerous stage-specific OTUs and a substantial shared core between the vegetative and reproductive phases (Figure 7B,C).

### 2.5. Functional Potential of Phyllosphere Microbiomes Differs by Resistance Class

To assess whether the observed taxonomic differences translate into functional variation, we used PICRUSt2 to predict metagenome functions from 16S rRNA data. In the resistant varieties, Metabolism was the dominant category across all resistance groups (Supplementary Figure S11). However, notable gradients were observed: Metabolism remained the dominant functional category across all resistance groups, showing a slight increase from resistant (48%) to susceptible varieties (50%). Conversely, Genetic Information Processing decreased along the same gradient (25% → 24% → 23%), as did Environmental Information Processing (18% → 17% → 16%). Unclassified functions were lowest in resistant varieties (10%) and highest in susceptible varieties (16%), suggesting that susceptible-associated microbiomes harbor a greater proportion of functionally uncharacterized taxa.

In the mid-resistant varieties, Translation emerged as the most abundant functional category, comprising 25% of predicted functions across all resistance groups, followed by Membrane Transport at 15% (Supplementary Figure S12). The most striking pattern was observed in Metabolism of Cofactors and Vitamins, which showed a progressive three-fold increase from resistant (5%) to mid-tolerant (10%) to susceptible (15%) varieties. This gradient suggests a potential link between cofactor metabolism and disease susceptibility. Poorly characterized functions followed the same pattern (5% → 10% → 15%), consistent with the Level 1 observation of increased unclassified functions in susceptible varieties.

KEGG analysis in the susceptible varieties revealed specific pathway-level differences (Supplementary Figure S13). DNA repair and recombination proteins showed striking enrichment in mid-tolerant varieties (>10%) compared to resistant (5%) and susceptible (5%) varieties, suggesting that microbiomes associated with moderately resistant genotypes may possess enhanced genomic maintenance and stress response capabilities. Photosynthesis-related pathways showed elevated levels in susceptible varieties, consistent with the taxonomic observation of increased Cyanobacteria in these varieties (Supplementary Figure S1). Purine metabolism was slightly higher in resistant varieties (5%) compared to mid-tolerant and susceptible varieties (<10%).

These functional predictions complement the taxonomic findings and suggest that resistance-associated microbiomes differ not only in composition but also in metabolic potential.

### 2.6. Isolation and Identification of Antagonistic Burkholderia from Resistant Cultivars

Approximately 148 endophytes and 133 epiphytes were isolated from rice plant tissues and evaluated for their capacity to inhibit *Rhizoctonia solani* AG1-IA. Thirty endophytes had only weak, sporadic effects on inhibiting the fungal growth and only four *Burkholderia* species inhibited the growth of the fungus consistently with a duration of longer than one month in the co-culture bioassays.

Among all the endophytes studied, 127 were Gram-negative, while 13 were Gram-positive. Further testing for functional properties indicated that 47 endophytes had the ability to fix nitrogen (see Supplementary Figure S7), and their additional bio-chemical properties (such as antibiotic resistance) can be found in Supplementary File S3.

Phylogenetic analysis based on the 16S rRNA sequences of the isolated bacteria confirmed that the bacteria isolated were of the genus *Burkholderia*. Furthermore, *B. vietnamiensis* was shown through this phylogenetic analysis to have a separate branch than known pathogenic species of *Burkholderia* such as *B. gladioli* and *B. cepacia* (Supplementary Figure S8). Therefore, *B. vietnamiensis* is classified as being within the non-pathogenic group.

### 2.7. Burkholderia Strains Exhibit Potent Antifungal Activity Against R. solani

**Figure 8:**
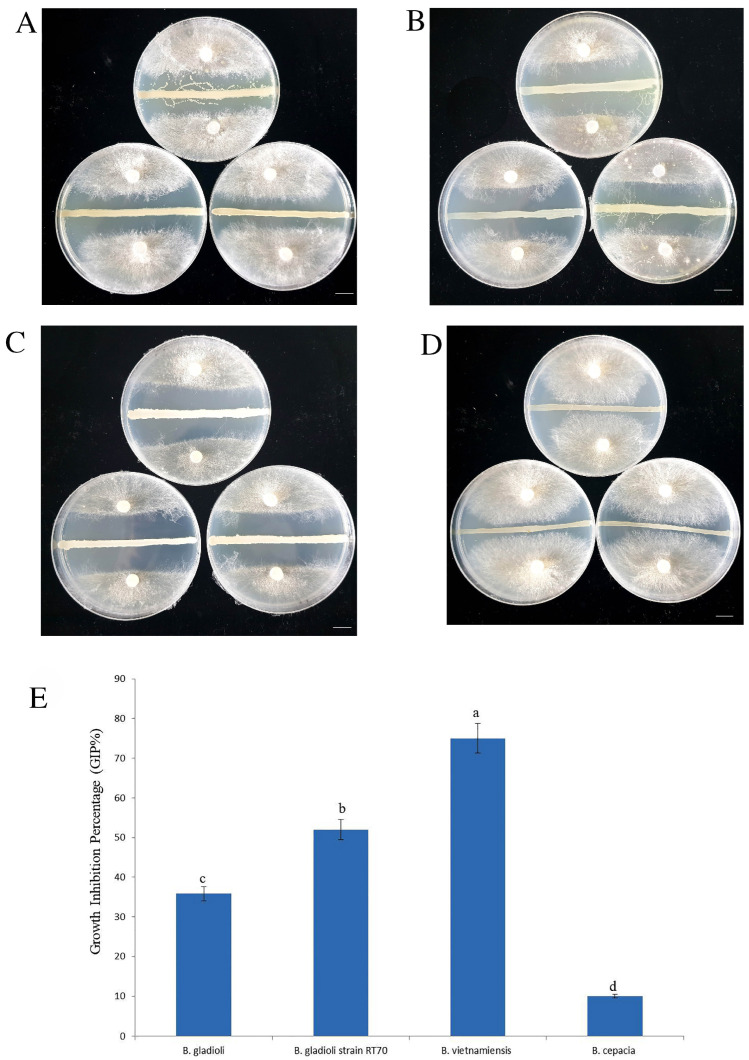
Antifungal activity of *Burkholderia* strains against *R. solani*. Dual culture assays performed on Potato Dextrose Agar (PDA) showing growth inhibition of *R. solani* by (**A**) *B. gladioli*, (**B**) *B. gladioli* strain A1EPSEED5, (**C**) *B. vietnamiensis*, and (**D**) *B. cepacia*. (**E**) Bars represent mean growth inhibition percentages. Different lowercase letters above the bars indicate statistically significant differences between treatments (*p* < 0.05).

Antifungal activities of the chosen *Burkholderia* strains against *R. solani* AG1IA are depicted in Figure 8. The percent GIP was determined using dual culture assay at 14 dpi. As demonstrated in Figure 8, *B. vietnamiensis* J14EPLEAF2 produced the greatest antifungal effect with a GIP value of 77%, while GIP values for the remaining strains were as follows: *B. gladioli* A1EPSEED5, 52%; *B. gladioli*, 36%; and *B. cepacia* J14Eple, 10%.

**Figure 9:**
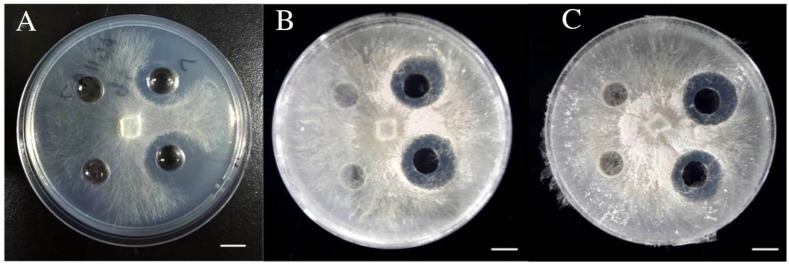
Antifungal activity of intracellular metabolites produced by *Burkholderia* against *R. solani*. Agar well diffusion assay showing inhibition of fungal growth by intracellular metabolite extracts at different incubation times: (**A**) after 2 days, (**B**) after 7 days, and (**C**) after 14 days. Clear inhibition zones surrounding the wells indicate time-dependent antifungal activity of intracellular metabolites.

Figure 9 also demonstrates that intracellular metabolites from *B. vietnamiensis* J14EPLEAF2 inhibited the growth of *R. solani* AG1IA. Therefore, it appears that *B. vietnamiensis* J14Epleaf2 is the most effective of the tested strains as an antagonist to *R. solani*.

**Figure 10:**
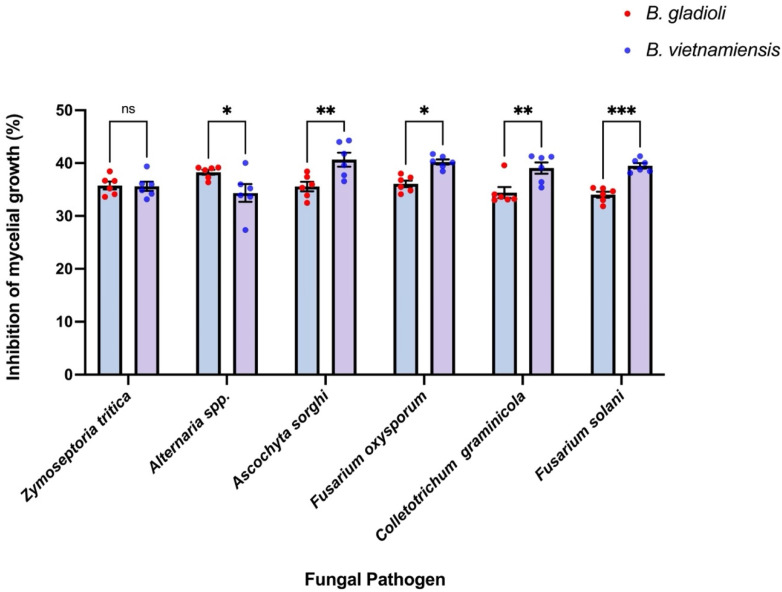
Inhibition of mycelial growth by two lead *Burkholderia* strains against diverse fungal pathogens. Bar graph showing the percentage inhibition of mycelial growth for six phytopathogenic fungi in dual-culture assays with *B. gladioli* A1EPSEED5 (red dots) and *B. vietnamiensis* J14EPLEAF2 (blue dots). Each bar represents the mean inhibition percentage ± standard error (n = 5). Statistically significant differences between the two strains for each pathogen were determined using unpaired *t*-tests: *p* < 0.05 (*), *p* < 0.01 (**), *p* < 0.001 (***), and not significant (ns).

**Figure 11:**
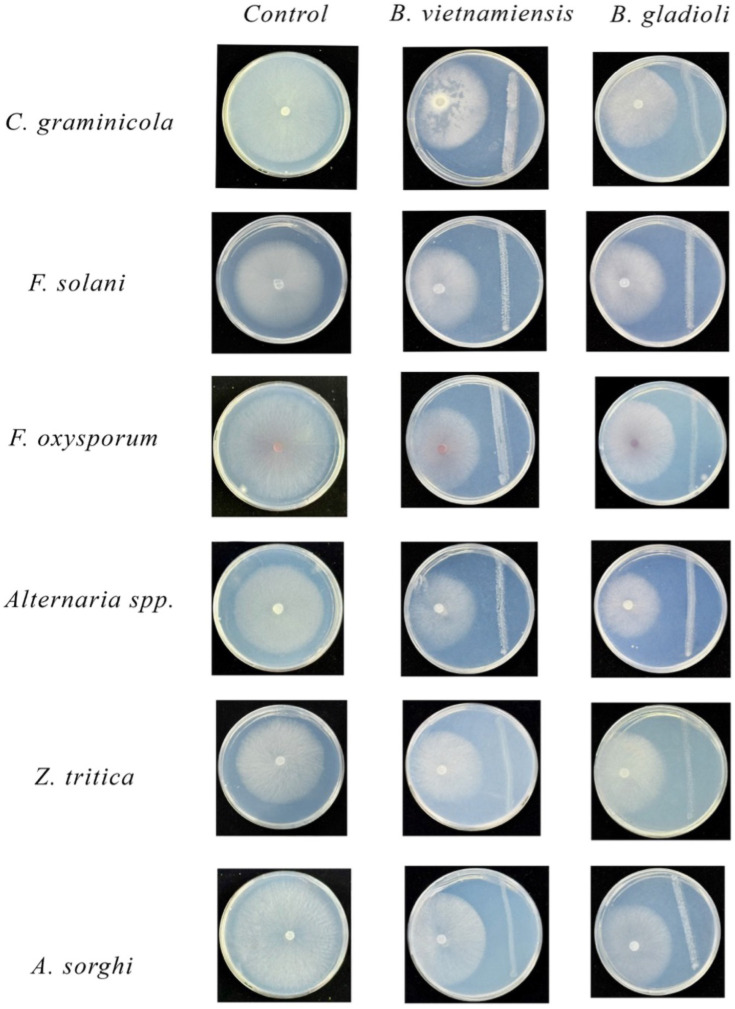
Representative dual-culture assay plates showing fungal growth inhibition by *B. vietnamiensis* J14EPLEAF2 and *B. gladioli* A1EPSEED5. Each row corresponds to a different fungal pathogen, with columns showing the control (fungus alone), fungus co-cultured with *B. vietnamiensis* J14EPLEAF2, and fungus co-cultured with *B. gladioli* A1EPSEED5.

The antifungal activities of *B. vietnamiensis* J14EPLEAF2 and *B. gladioli* A1EPSEED5 were tested in comparison to each other as well as six additional phytopathogens using dual culture testing methods. In both comparisons, *B. vietnamiensis* J14EPLEAF2 and *B. gladioli* A1EPSEED5 were shown to be able to suppress the mycelial development of all of the pathogens that were evaluated, thereby demonstrating a broad spectrum of antagonism. However, *B. vietnamiensis* J14EPLEAF2 was shown to have greater suppression of pathogen mycelial development than *B. gladioli* A1EPSEED5 at an average rate of 25% when compared to the percentage of mycelial development of the same pathogens suppressed by *B. gladioli* A1EPSEED5. Specifically, *B. vietnamiensis* J14EPLEAF2 had significantly higher levels of suppression for the pathogens *Ascochyta sorghi*, *Fusarium oxysporum*, *Colletotrichum graminicola* and *Fusarium solani* when compared to *B. gladioli* A1EPSEED5 (Figure 10 and Figure 11).

### 2.8. Burkholderia Treatments Suppress Sheath Blight Development and Sclerotia Formation

**Figure 12:**
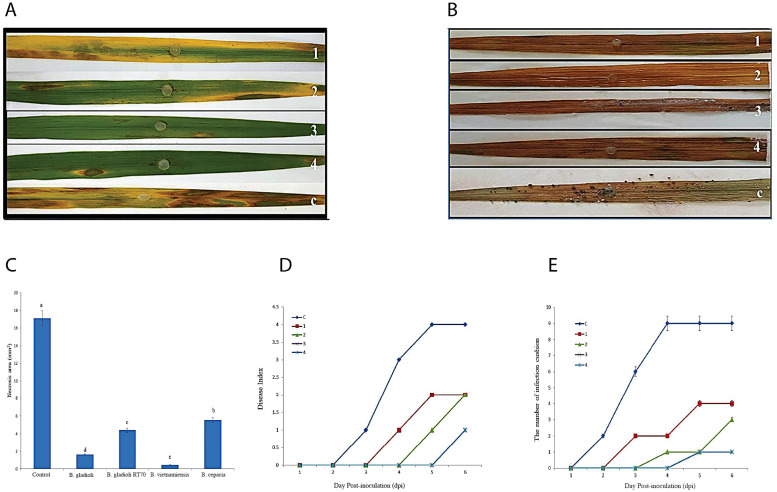
Effect of beneficial bacteria on the progression of sheath blight in rice (cv. Lemont). (**A**) Disease progression on 4-month-old rice leaves treated with beneficial bacteria (1. *B. cepacia*, 2. *B. gladioli* strain A1EPSEED5, 3. *B. vietnamiensis*, 4. *B. gladioli* RT70) and a control (0.05% Tween 20) at 4 days post inoculation with R. solani AG1IA. (**B**) Effect of bacterial treatments on sclerotia formation on rice plant debris. (**C**) Quantification of necrotic areas using ImageJ software (Version 1.6.2-24). (**D**) Disease progression from 0 to 6 days post inoculation on rice leaves with or without bacterial treatment. (**E**) Number of infection cushions formed in the presence or absence of beneficial bacteria. Statistical significance was determined using Duncan’s multiple range test at *p* < 0.05. Means with different letters indicate significant differences.

Treatment of rice leaves with *Burkholderia* species resulted in a significant decrease in disease progression and a substantial reduction in the disease index caused by *R. solani* AG1IA compared with the untreated control. Among the tested strains, *B. vietnamiensis* was the most effective in reducing necrosis symptoms (Figure 12A,C,D). Sclerotia formation was observed in control leaves beginning at 4 days post inoculation (dpi), and after one week, 18–20 sclerotia per leaf were recorded. In contrast, sclerotia were rarely formed on leaves treated with *Burkholderia* species, indicating strong suppression of fungal reproductive structures (Figure 12B).

The infection process of *R. solani* AG1IA was compared on rice leaves in the presence and absence of *Burkholderia* species. Observation of infection cushions at 2, 3, 4, 5, and 6 dpi revealed that their formation was delayed in leaves treated with *Burkholderia* strains. In contrast, control leaves without bacterial treatment showed a high level of infection cushions by 4 dpi (Figure 12E), indicating that *Burkholderia* species can effectively slow early fungal infection.

### 2.9. Field and Greenhouse Evaluation of Burkholderia Strains Against Sheath Blight

**Figure 13:**
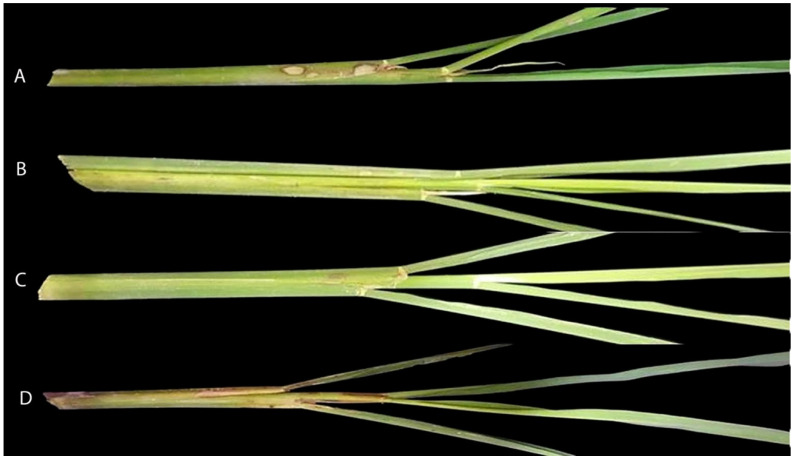
Sheath blight lesions on rice plants inoculated with Rhizoctonia solani AG1IA in the presence and absence of Burkholderia species under field conditions. (**A**) Rice plant treated with *B. gladioli*, (**B**) Rice plant treated with *B. cepacia*, (**C**) Rice plant treated with B. vietnamiensis, (**D**) control (0.05% Tween 20).

To validate the results obtained from laboratory and greenhouse experiments, the efficacy of *Burkholderia* strains was evaluated under natural field conditions. Symptoms of sheath blight appeared in control plants two days after inoculation. In contrast, plants treated with *Burkholderia* species showed no visible symptoms at the same time point, demonstrating effective disease suppression under field conditions (Figure 13).

### 2.10. Biosafety Assessment Distinguishes Pathogenic from Non-Pathogenic Strains

**Figure 14:**
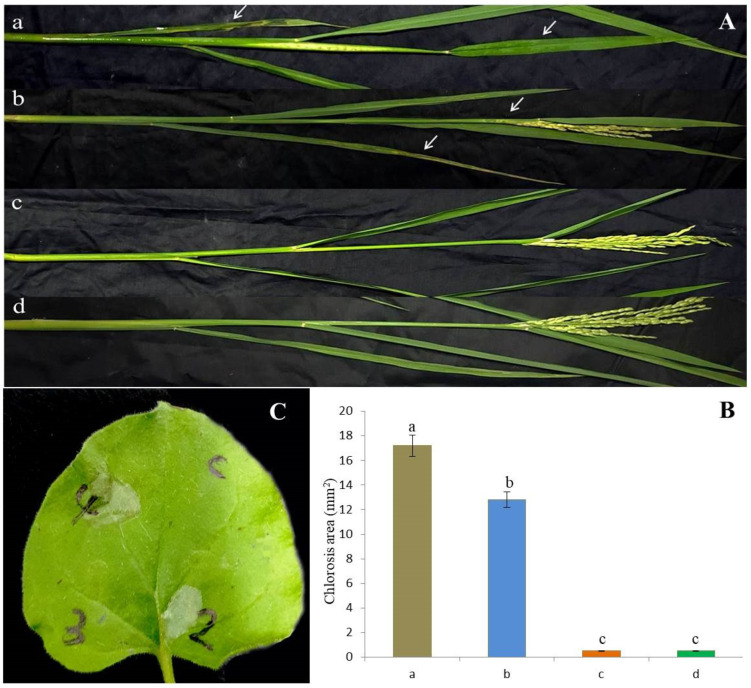
Hypersensitive response and pathogenicity of rice phyllosphere-derived Burkholderia species on tobacco and rice. (**A**) Pathogenicity assay on rice leaves infiltrated with bacterial suspensions (10^7^ CFU/mL in 0.05% Tween 20): a: *B. gladioli* strain A12Epseed5, b: *B. cepacia* strain J14Eple, c: *B. vietnamiensis* strain J14Epleaf2, d: control (0.05% Tween 20 in ddH_2_O). (**B**) Quantification of chlorosis area using ImageJ software. Different letters above bars indicate statistically significant differences according to Duncan’s multiple range test (*p* < 0.05). (**C**) Hypersensitive response (HR) in tobacco leaves at 1 day post infiltration with bacterial suspensions (10^7^ CFU/mL): 1: ddH_2_O control, 2: *B. cepacia* strain J14Eple, 3: *B. vietnamiensis* strain J14Epleaf2, 4: *B. gladioli* strain A12Epseed5.

Hypersensitive response (HR) assays on tobacco (*Nicotiana benthamiana*) and pathogenicity tests on rice clearly differentiated non-pathogenic from pathogenic strains. Leaf tissues infiltrated with *B. gladioli* A12Epseed5 or *B. cepacia* J14Eple developed necrotic lesions within 24 h, while areas infiltrated with *B. vietnamiensis* J14Epleaf2 or sterile water remained healthy for up to one week (Figure 14). On whole rice plants, pathogenic strains caused chlorosis and water-soaked lesions on blades, panicles, and sheaths within 3 days, whereas *B. vietnamiensis*-treated plants remained symptomless (Figure 14). These results establish *B. vietnamiensis* as a safe biocontrol candidate with no detectable pathogenic potential toward rice or tobacco.

### 2.11. Burkholderia Strains Possess Multiple Plant Growth-Promoting Traits

#### 2.11.1. Amylase Activity Assay

**Figure 15:**
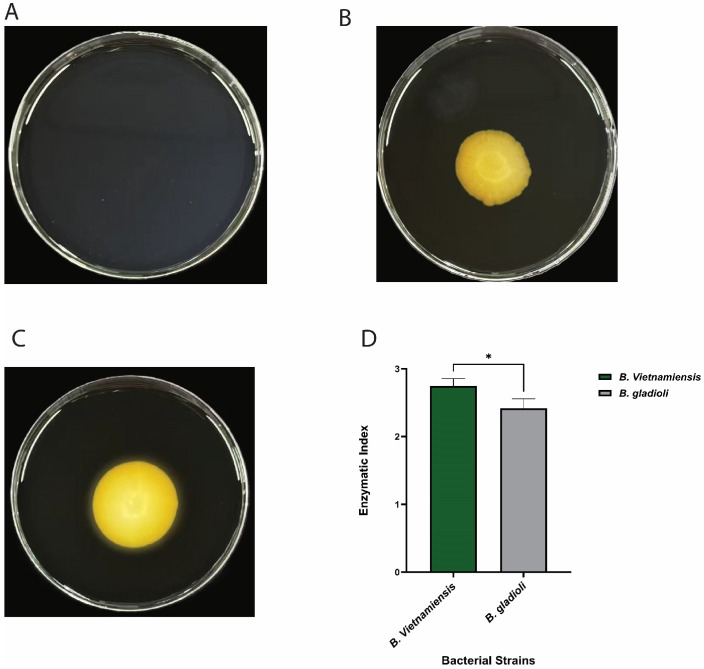
Extracellular amylase activity in *B. vietnamiensis* and *B. gladioli* strains. Starch hydrolysis assay on agar plates showing clear halo zones indicative of amylase activity. (**A**) Uninoculated control. (**B**) Burkholderia gladioli. (**C**) Burkholderia vietnamiensis. (**D**) Comparison of Enzymatic Index (EI) values between *B. vietnamiensis* and *B. gladioli*. Bars represent mean ± standard error. Asterisks (*) indicate a statistically significant difference at *p* < 0.05.

Both bacterial strains produced amylase, as evidenced by distinct, clear halo zones surrounding colonies on starch agar plates (Figure 15). *B. vietnamiensis* exhibited significantly higher amylase activity than *B. gladioli* (*p* < 0.05). The mean enzymatic index (EI) value for *B. vietnamiensis* was approximately 2.75, while *B. gladioli* showed a significantly lower mean EI of approximately 2.40.

#### 2.11.2. Siderophore Production

**Figure 16:**
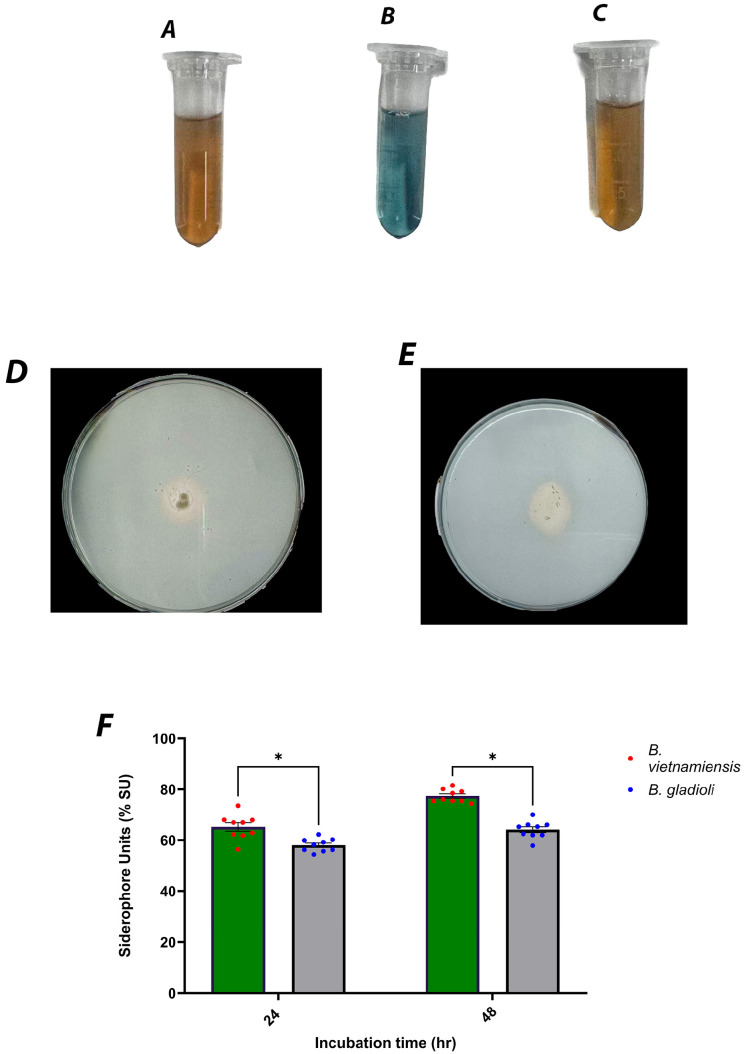
Detection and quantification of siderophore production by Burkholderia species. (**A**–**C**) Qualitative liquid assay using CAS reagent: (**A**) Burkholderia vietnamiensis supernatant showing orange color (positive reaction); (**B**) Control (blue, negative reaction); (**C**) *Burkholderia gladioli* supernatant showing orange color (positive reaction). (**D**,**E**) Qualitative plate assay on CAS agar: (**D**) *B. gladioli* exhibiting a hydrolysis halo; (**E**) *B. vietnamiensis* exhibiting a distinct hydrolysis halo. (**F**) Quantitative comparison of siderophore production expressed as percentage Siderophore Units (% SU) at 24 and 48 h post inoculation. Green bars represent B. vietnamiensis; gray bars represent *B. gladioli*. Error bars indicate standard error. Asterisks (*) denote statistically significant differences (*p* * < 0.05).

The liquid CAS assay resulted in a clear color change of the CAS reagent from blue to yellow, which is indicative of siderophore-mediated iron binding (Figure 16A–C), as well as on CAS agar plates, both *B. gladioli* and *B. vietnamiensis* had visible haloes of CAS activity around their colonies (Figure 16D,E). The quantitative data showed an increase in siderophore production over time for both strains (Figure 16F), with *B. vietnamiensis* at 24 h and 48 h producing significantly more siderophores than *B. gladioli* (*p* < 0.05).

#### 2.11.3. Indole-3-Acetic Acid (IAA) Production

**Figure 17:**
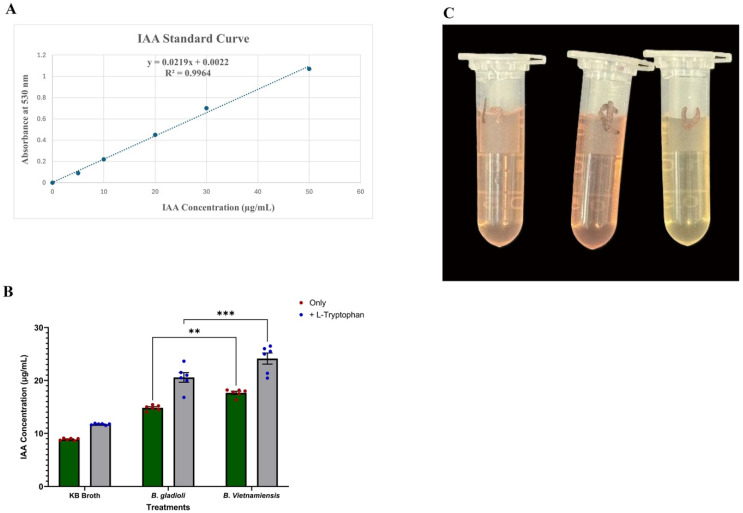
Indole-3-acetic acid (IAA) production by Burkholderia strains and its quantification using the Salkowski assay. (**A**) Standard curve for IAA quantification generated using known concentrations of indole-3-acetic acid. Absorbance was measured at 530 nm. The linear regression equation (y = 0.0219x + 0.0022) and high coefficient of determination (R^2^ = 0.9964) indicate a strong linear relationship between IAA concentration and absorbance. (**B**) IAA production by Burkholderia gladioli and Burkholderia vietnamiensis cultured in KB broth with and without L-tryptophan supplementation. Bars represent mean ± standard error (SE). Statistical significance is indicated by asterisks (** *p* < 0.01; *** *p* < 0.001). (**C**) Representative visual appearance of the Salkowski assay. Development of pink coloration indicates IAA presence.

The amount of Indole Acetic Acid (IAA) produced by both strains was evaluated using the Salkowski assay (Figure 17). Compared to uninoculated control samples, both strains produced significantly more IAA; however, when L-tryptophan was added to the growth media as a precursor for tryptophan to be converted into IAM (indole-3-acetamide) for conversion to IAA, *B. vietnamiensis* had a greater base level of IAA production that increased in response to L-tryptophan addition (*p* < 0.001) compared to *B. gladioli* (*p* < 0.01) (Figure 17B,C), thus *B. vietnamiensis* has an enhanced ability to produce IAA via the IAM pathway.

#### 2.11.4. Phosphate Solubilization

**Figure 18:**
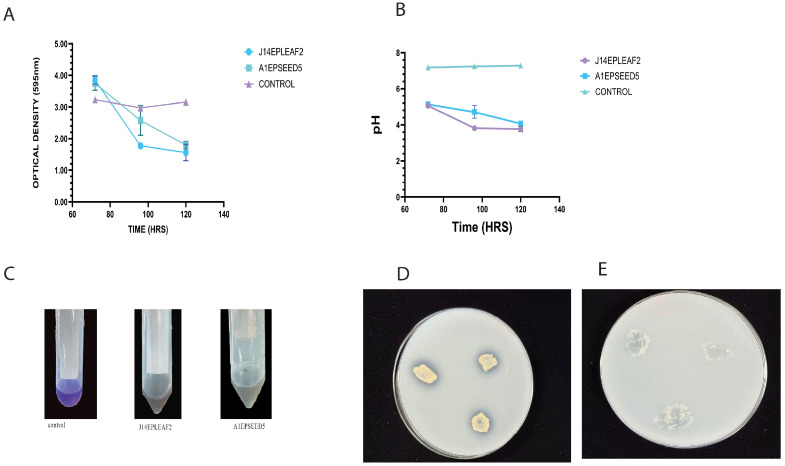
Phosphate solubilization by *Burkholderia* strains. (**A**) Changes in bromophenol blue (BPB) absorbance at 595 nm (OD_595_) over time in BPB–NBRIP broth inoculated with *B. vietnamiensis* (J14EPLEAF2), *B. gladioli* (A1EPSEED5), and an uninoculated control. Decreasing OD_595_ values indicate BPB color transition associated with medium acidification during phosphate solubilization. (**B**) Corresponding changes in culture medium pH over time. Both bacterial strains caused a progressive decrease in pH, whereas the control medium remained relatively stable throughout the incubation period. (**C**) Representative photographs of BPB–NBRIP broth after incubation. The Control uninoculated with no added bacteria retained its original purple colored BPB, while all cultures that were inoculated with either J14EPLEAF2 and/or A1EPSEED5 had an obvious color change that indicated the bacteria were able to acidify BPB. (**D**,**E**) Solid phosphate medium qualitative assessment for phosphate solubilization by both *B. vietnamiensis* (**D**) and *B. gladioli* (**E**). Both exhibited an obvious clearing zone in the area surrounding the bacterial colonies where they grew, as well as a change in transparency/opacity, clearly demonstrating phosphate solubilization from the insoluble phosphate in the medium.

Both *Burkholderia* strains displayed phosphate solubilization in both solid and liquid media. In BPB-NBRIP broth, both strains acidified the broth, reducing absorbance (OD595) over time compared to the control culture (Figure 18A). Absorbance (OD595) decreased at a greater rate in cultures inoculated with *B. vietnamiensis* than in cultures inoculated with *B. gladioli*. The pH of the medium decreased significantly in cultures treated with either bacterium, whereas the control culture did not (Figure 18B). *B. vietnamiensis* reduced the pH of the broth at a greater and more rapid rate than *B. gladioli*. Visible evidence of phosphate solubilization occurred when the broth was inoculated with both bacteria, since the two cultures exhibited a visible change (Figure 18C–E).

#### 2.11.5. Proteolytic Activity

**Figure 19:**
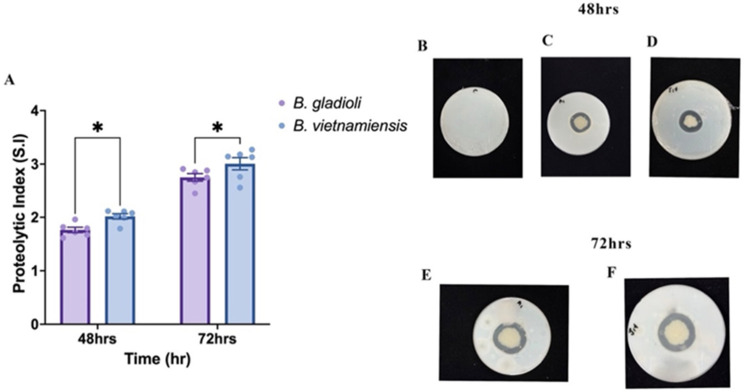
Extracellular proteolytic activity of Burkholderia strains on skim milk agar. (**A**) Quantitative comparison of proteolytic activity expressed as proteolytic index (PI) for *B. gladioli* and *B. vietnamiensis* after 48 and 72 h of incubation. Bars represent mean ± standard error (SE) of biological replicates. Asterisks indicate significant differences between strains at the same time point (*p* < 0.05). (**B**) Uninoculated skim milk agar plate showing no casein hydrolysis (negative control). (**C**) Skim milk agar plate inoculated with *B. gladioli* after 48 h of incubation, showing a clear halo zone surrounding the bacterial colony, indicative of extracellular protease activity. (**D**) Skim milk agar plate inoculated with *B. vietnamiensis* after 48 h of incubation, showing a distinct zone of casein hydrolysis. (**E**) Skim milk agar plate inoculated with *B. gladioli* after 72 h of incubation, exhibiting an enlarged proteolytic halo compared to 48 h. (**F**) Skim milk agar plate inoculated with *B. vietnamiensis* after 72 h of incubation, showing a pronounced zone of casein hydrolysis consistent with increased protease activity.

At both time points of 48 h and 72 h, there were observable halos of enzymatic degradation on skim milk agar plates, as evidenced by each of the isolates tested (see Figure 19C–F), which indicated that both isolates produced proteolytic activity. For *B. vietnamiensis*, the proteolytic index significantly increased (*p* < 0.05) over that of *B. gladioli* after both 48 h and 72 h incubation periods. The amount of enzymatic degradation also increased for both isolates, as measured by the proteolytic index (Figure 19A).

#### 2.11.6. Seed Germination and Seedling Growth

**Figure 20:**
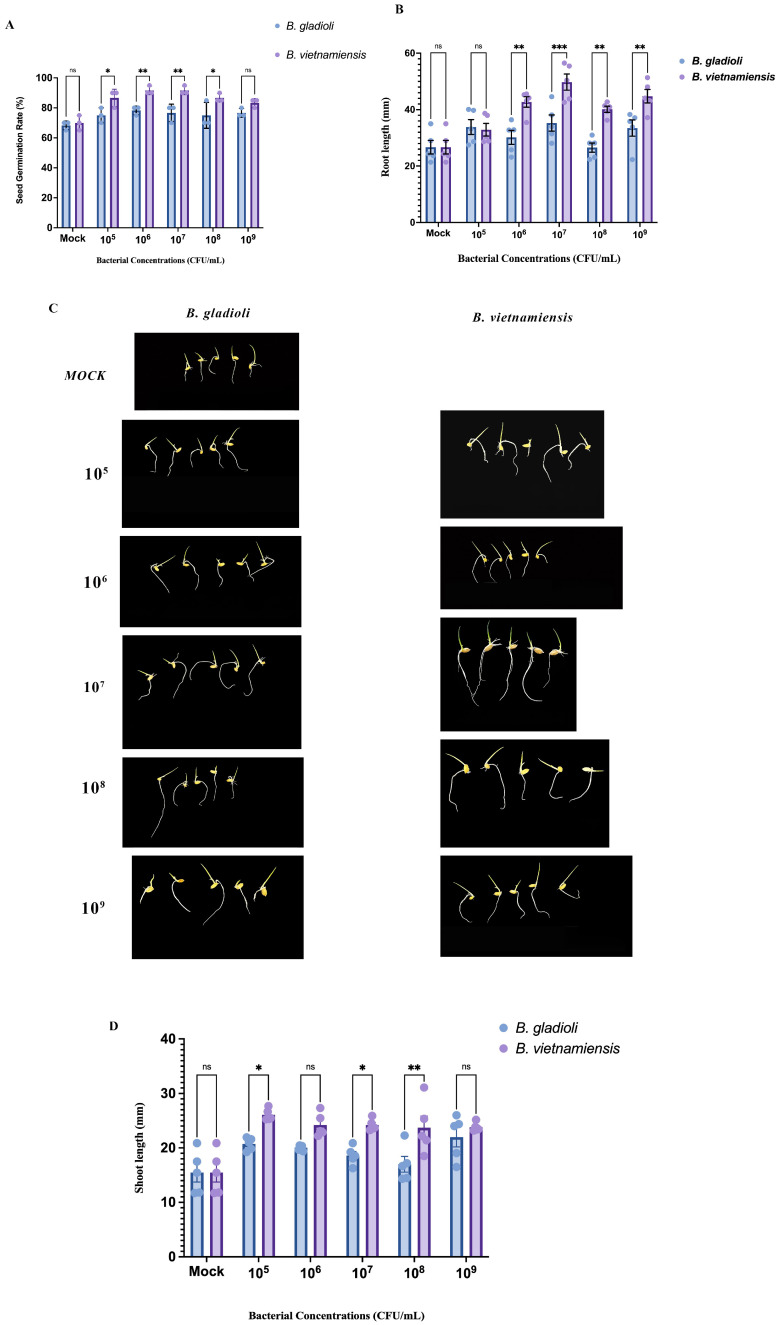
Effects of *Burkholderia* strains on seed germination and early seedling growth of rice (cv. Zhonghua 11). (**A**) Seed germination rate (%) following treatment with *Burkholderia gladioli* and *Burkholderia vietnamiensis* at different bacterial concentrations (10^5^–10^9^ CFU mL^−1^). (**B**) Root length (mm) of rice seedlings under the same treatments. (**C**) Representative images of germinated rice seedlings treated with *B. gladioli* (**left**) and *B. vietnamiensis* (**right**) at indicated bacterial concentrations; mock-treated seeds served as controls. (**D**) Shoot length (mm) of rice seedlings following bacterial treatments. Bars represent mean ± SEM of three independent biological replicates. Statistical significance between bacterial strains at the same concentration is indicated as ns (not significant), *p* < 0.05 (*), *p* < 0.01 (**), and *p* < 0.001 (***).

Both *Burkholderia* strains influenced rice seed germination in a dose-dependent manner (Figure 20). *Burkholderia vietnamiensis* was positive for germination at all tested concentrations (10^6^–10^7^ CFU mL^−1^), where germination heightened significantly at 10^7^ CFU mL^−1^ (*p* < 0.01). The root growth was measured by measuring the length of the roots after treatment with both *Burkholderia* strains. From 10^5^ CFU mL^−1^ onwards, *B. vietnamiensis* induced a greater root elongation than *B. gladioli*. With regard to the elongation of the roots, the most noticeable effects were observed at 10^7^ CFU mL^−1^ (*p* < 0.001). Shoot length analysis showed that *B. vietnamiensis* caused a significant increase at 10^5^ and 10^7^ CFU mL^−1^ (*p* < 0.05).

### 2.12. Burkholderia Vietnamiensis Primes Defense-Related Gene Expression

The expression levels of PR10 (salicylic acid pathway marker) and PR5 (jasmonic acid pathway marker) were evaluated following inoculation with *B. vietnamiensis*. In both resistant and susceptible rice cultivars, inoculation with *B. vietnamiensis* alone resulted in modest but non-significant alteration in PR10 and PR5 transcript levels compared with untreated controls (*p* > 0.05).

**Figure 21:**
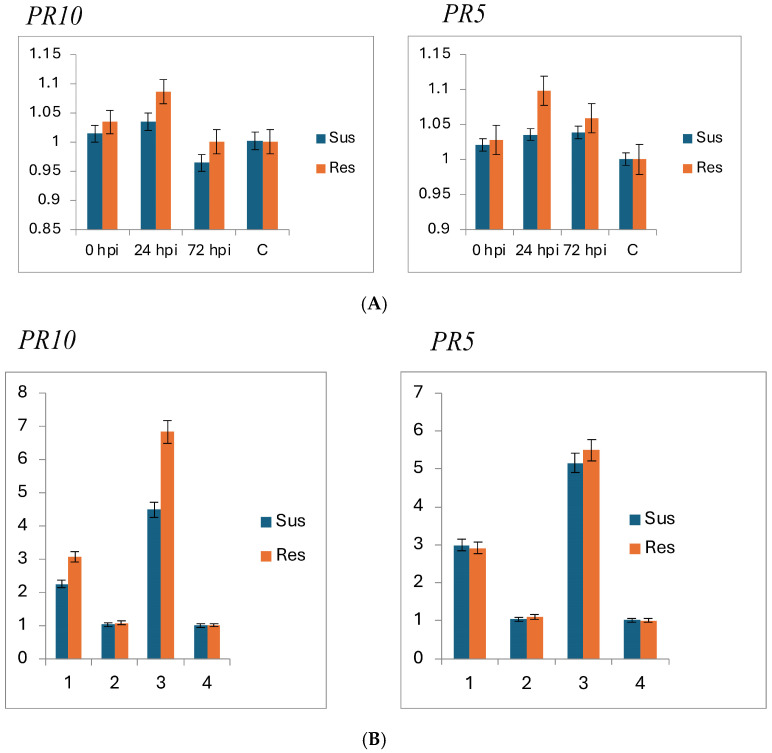
Expression pattern of PR genes post inoculation with *Burkholderia vietnamiensis* in resistance and susceptible rice varieties (**A**). Expression pattern of SA- (PR5) and JA- (PR10) in three different time points post inoculation with *Burkholderia vietnamiensis* in resistance and susceptible rice varieties. Quantitative real-time PCR analysis of PR gene expression at 0, 24, and 72 h post inoculation (hpi) in susceptible (CT18233-15-3-3-4-1-1) and resistant (IR57301-158-3-1) rice varieties. Values represent fold change relative to mock-inoculated controls at each time point. Statistical analysis was performed using a completely randomized design (CRD), and means were separated using Duncan’s multiple-range test at *p* ≤ 0.05. Error bars represent standard deviation (SD) from three biological replicates with two technical repeats each. (**B**). Expression of SA—(PR5) and JA—(PR10) in rice seedlings treated with *Burkholderia vietnamiensis* and challenged with *Rhizoctonia solani*. Relative expression levels of PR10 and PR5 genes in three-week-old rice seedlings of susceptible (CT18233-15-3-3-4-1-1) and resistant (IR57301-158-3-1) varieties under different treatment conditions: 1 = *R. solani* AG1IA alone, 2 = *B. vietnamiensis* alone, 3 = *B. vietnamiensis* + R. solani AG1IA (co-inoculation), and 4 = untreated control. Gene expression was quantified by real-time PCR and normalized to untreated controls. Statistical analysis was performed using a completely randomized design (CRD), and means were separated using Duncan’s multiple-range test at *p* ≤ 0.05. Error bars represent standard deviation (SD) from three biological replicates with two technical repeats each.

To evaluate priming effects, rice seedlings were challenged with *R. solani* AG1IA 3 days after bacterial inoculation. Upon pathogen infection, seedlings pre-treated with *B. vietnamiensis* exhibited significantly higher expression levels of PR10 and PR5 compared to seedlings inoculated with *R. solani* alone (*p* < 0.05) (Figure 21A,B). In both resistant and susceptible cultivars, *R. solani* induced upregulation of PR10 and PR5, but expression levels were much higher in seedlings pre-treated with *B. vietnamiensis* prior to pathogen exposure.

### 2.13. LC-MS Metabolite Profiling

**Figure 22:**
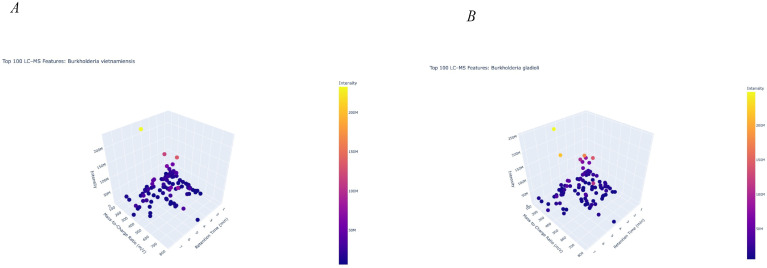
Comparative LC-MS metabolite profiling of *Burkholderia species.* (**A**) Top 100 LC-MS features from *Burkholderia vietnamiensis* culture extracts, plotted by intensity. (**B**) Top 100 LC-MS features from *Burkholderia gladioli* culture extracts. Intensity values (0–200 M) represent relative abundance of detected metabolites. The distinct metabolite profiles reflect species-specific metabolic capabilities and potential bioactivity.

LC–MS analysis of *Burkholderia vietnamiensis* culture extracts revealed a diverse set of secondary metabolites (Figure 22A). Among the detected compounds was N5-formyl-N5-hydroxyornithine (*m*/*z* = 176.1029, rt = 0.86 min), an intermediate involved in the biosynthesis of the siderophore ornibactin, suggesting a potential role in iron acquisition and competitive exclusion of fungal pathogens. In addition, three N-acyl homoserine lactones (AHLs)—N-butyryl-L-homoserine lactone (C4-HSL) (*m*/*z* = 172.0968, rt = 1.52 min), N-decanoyl-L-homoserine lactone (C10-HSL) (*m*/*z* = 272.1869, rt = 6.09 min), and N-dodecanoyl-L-homoserine lactone (C12-HSL) (*m*/*z* = 314.2343, rt = 6.81 min)—were identified. These molecules are well-known quorum sensing signals in *Burkholderia* spp. and may regulate the coordinated production of antifungal metabolites and biofilm formation. Indole-3-acetic acid (IAA) (*m*/*z* = 174.0564, rt = 5.24 min) was also detected, consistent with previously reported plant growth-promoting traits of *Burkholderia* species. While these metabolites are associated with antimicrobial activity and microbial signaling, the specific compound(s) responsible for the observed 77% inhibition of *Rhizoctonia solani* cannot be definitively determined from LC–MS profiling alone. It is therefore likely that antifungal activity results from combined or synergistic effects of multiple metabolite classes, including siderophore-mediated competition, quorum sensing-regulated secondary metabolism, and other bioactive compounds produced by the strain.

*B. gladioli* had a different pattern of distribution for the peak intensity of metabolites (Figure 22B). The highest amount of metabolites had higher peak intensities than *B. vietnamiensis* at *m*/*z* values of 300–500 which includes moiramide A/B/C, sevadicin, and malformin B2.

## 3. Discussion

The current study analyzed the phyllosphere microbiome of 100 rice cultivars differing in their susceptibility to sheath blight disease caused by Rhizoctonia solani AG1IA. Data from this investigation indicate that microbial communities of rice varieties with high, moderate, or low levels of resistance to the disease exhibit unique structural characteristics. These results suggest that the plant host genotype may play an important role in shaping unique populations of bacteria on leaves (phyllosphere) and that these populations may be linked to disease development or control.

### 3.1. Phyllosphere Microbiome Composition and Disease Resistance

The results from this study show that the rice phyllosphere at the tillering stage contains a large number of bacteria belonging to 34 different phyla, and the most abundant include *Cyanobacteria*, *Proteobacteria*, *Firmicutes*, *Bacteroidota*, and *Actinobacteriota* (see Supplementary File S4). This taxonomic composition is generally similar to that found for rice phyllosphere communities in recent reviews and meta-analyses of rice phyllosphere microbiomes that have identified *Proteobacteria*, *Actinobacteria* and *Bacteroidetes* as core phyla [22], and have also noted that Firmicutes and Cyanobacteria are commonly found on rice leaves. Additionally, the finding of a large number of minor phyla in resistant and mid-resistant rice varieties (such as *Myxococcota*, *Chloroflexi*, *Gemmatimonadota*, *Nitrospirota*, and *Planctomycota*) indicates that the microbial community structure in disease-resistant rice genotypes may be both phylogenetically more diverse and functionally more heterogeneous than those found in non-resistant (susceptible) varieties [22].

At the family level, resistant and tolerant varieties showed enrichment in *Burkholderiaceae*, *Bacillaceae*, *Pseudomonadaceae*, and *Oxalobacteraceae*, whereas susceptible varieties exhibited higher relative abundances of *Sphingobacteriaceae*, *Enterobacteriaceae*, and *Staphylococcaceae* (Figure 2), consistent with previous studies [16]. Many of the families enriched in resistant lines contain taxa with documented plant-growth-promoting or biocontrol functions in rice, particularly *Burkholderiaceae*, *Bacillaceae*, and *Pseudomonadaceae*, which include strains that suppress *R. solani* and other rice pathogens through antifungal metabolites, competition, and induction of systemic resistance [23].

The taxonomic shift in genotypes observed in this study was also reflected in the predicted functional profiles of each plant variety’s microbiome (resistance-associated vs. susceptible), indicating that a plant’s microbiome can have different metabolic capacities than the same microbe when it is found in a plant lacking resistance to pathogens. Picrust2 analysis predicted that susceptible plant varieties harbor an overabundance of microbiomes involved in photosynthesis, such as cyanobacteria [24], which is expected given the high photosynthetic activity on the leaves of susceptible plants. The high levels of Metabolism of Cofactors and Vitamins in the microbiomes associated with susceptible plant varieties may indicate that the microbiome of susceptible plants provides additional nutritional support for the pathogen as it colonizes the host plant [25] or may result from changes in nutrient availability due to pathogen infection. In contrast, the presence of DNA repair and recombination protein genes in the microbiomes of mid-tolerant varieties may suggest that the microbiome of a mid-tolerant plant variety has increased capability for maintaining its genome; therefore, if it does get infected by a pathogen, it may be able to withstand that pathogen better than other plant varieties [26]. Additionally, the greater number of unclassified functions in the microbiomes of susceptible plant varieties indicates a significant lack of information about the microbial communities on those plants, suggesting that a large portion of the microbial population is functionally unknown.

### 3.2. Genotype-Specific Microbial Signatures and Divergence Among Resistance Classes

The multivariate analysis of OTU relative abundance data has revealed substantial compositional variation across the three resistance classes (Figure 1 and Figure 5). The results from the AMOVA test indicate there were statistically significant beta diversity differences between the susceptible and resistant groups as well as between the susceptible and mid-tolerant groups but there was significantly less difference between the resistant and mid-tolerant groups (Table 3), which is indicative that both the host genotype and the resistance phenotype drive the phyllosphere community structure to a much larger degree than do within group differences. A similar pattern has also been found by others studying other crops with different levels of resistance in their cultivars which was identified as the primary determinant of the unique microbial community present in those crops [23,27,28].

MicroPITA analysis identified that some resistant and mid-tolerant rice varieties exhibited “extreme” (dissimilar to others) or high levels of diversity of their phyllosphere microbial community. These results are interpreted as due either to host genetic influence on microbial community composition through filtering mechanisms or to differences in physiological traits between rice genotypes influencing microbial community assembly (Figure S9). This is consistent with earlier research demonstrating that rice genotype can be an overriding factor compared to other factors, such as domestication status, in structuring plant-associated microbial communities [29]. Mechanistic studies provide additional support for these interpretations by identifying allelic variations in important biochemical pathways (e.g., phenylpropanoid biosynthesis) that alter leaf metabolites, which, in turn, alter the relative abundance of certain phyllosphere microbial taxa [17].

### 3.3. Diversity–Disease Relationships and the Role of Core Taxa

The resistant rice genotypes used in this research had significantly greater numbers of bacteria and phylogenetic diversity than both tolerant and susceptible genotypes. These results are evident in all alpha-diversity metrics (Table 1 and Table 2; Figure 4) as well as from the substantially larger total and unique OTU numbers found in resistant rice (3230 OTUs/2064 Unique) compared to tolerant (1084 OTUs/135 Unique) and susceptible (599 OTUs/36 Unique) rice. The relationship between increased phyllosphere diversity and improved disease resistance is not limited to rice, however [30,31].

At the genus level, resistant and mid-tolerant varieties were enriched in *Bacillus*, *Pseudomonas*, and *Ralstonia*, whereas *Lactobacillus* and *Methylobacterium* were more prevalent in susceptible varieties (Figure 3), confirming previous findings [32]. Many *Bacillus* and *Pseudomonas* strains from the rice phyllosphere and rhizosphere are well-established biocontrol agents against sheath blight and other rice diseases [33]. Shrestha et al. [34] demonstrated that several rice-associated *Bacillus* spp. significantly reduced sheath blight severity in field trials, supporting the functional relevance of the *Bacillaceae* enrichment observed here in resistant genotypes.

### 3.4. Burkholderia vietnamiensis as a Potent Biocontrol Agent

Based on a large initial number of bacterial isolates, four *Burkholderia* strains were identified as strongly and stably inhibitory to *R. solani* in dual-culture tests. Of these, *B. vietnamiensis* J14EPLEAF2 and *B. gladioli* A1EPSEED5 were selected as promising strains due to their strong, stable inhibition of *R. solani*. The results of our experiments are in line with prior research findings indicating that rice-associated *Burkholderia* species have strong antagonistic properties toward *R. solani* [35,36] and that they exhibit high levels of inhibition, which suppresses mycelial development for a longer period than reported in prior research. Furthermore, the 77% inhibition displayed by *B. vietnamiensis* J14EPLEAF2 was significantly better than that reported in prior studies (64.8%) [37], including *B. pyrrocinia* S17-377.

*B. vietnamiensis* J14EPLEAF2 demonstrated a wide range of antimicrobial activity toward numerous plant pathogenic fungi (*Fusarium oxysporum*, *Ascochyta sorghi*, *Colletotrichum graminicola*, and *Fusarium solani*). These results support prior research demonstrating the polyphagous biocontrol activity of various *Burkholderia* species, including *Burkholderia C12* and *Burkholderia* sp. HQB-1 [38,39,40].

### 3.5. Disease Suppression Mechanisms

The results from detached-leaf, greenhouse, and field experiments clearly indicated that *Burkholderia* treatments significantly reduced sheath blight lesion development, delayed symptom expression, and nearly entirely eliminated sclerotia formation on rice leaves. The results were consistent with previous research that demonstrated significant suppression of *R. solani* infection and disease progression by leaf- and endophytic-associated *Burkholderia* [35,41].

Microscopic examination indicated that leaves treated with *Burkholderia* were less prone to forming infection cushions than untreated leaves. These results are consistent with previously described biocontrol mechanisms in which bacterial antagonists or their metabolites distort or collapse *R. solani* hyphae, interfere with sclerotia formation, and reduce the development of infection structures [37,42,43]. Such effects are often mediated by extracellular metabolites that disrupt fungal cell membranes, induce oxidative stress, or inhibit key developmental processes. As infection cushions represent primary enzymatically penetrable sites for *R. solani* [44], a plausible mechanism underlying the antagonistic activity of *Burkholderia*, *Bacillus*, and *Pseudomonas* spp. is the disruption of infection cushion formation or function [45,46]. In the present study, the detection of quorum-sensing molecules and siderophore-related metabolites further suggests that antifungal activity may be regulated through coordinated secondary metabolite production and competitive interactions, although the specific contribution of individual compounds cannot be conclusively determined without targeted validation assays.

Gene expression was used to determine that *B. vietnamiensis* primes for a defense response and significantly increases expression of PR5 and PR10 when challenged with pathogens; which is consistent with the findings of Esmaeel et al. [47], who studied the effects of the *Burkholderia* strains BE17 and BE24 on the relative transcript levels of pathogenesis related (PR) proteins *PR5* and *PR10* after infection with *Botrytis*. The primed state enables plants to respond to future infections faster and more strongly than non-primed plants, through SA- and JA-mediated signaling. These are examples of two mechanisms that represent important indirect ways in which plants suppress diseases.

### 3.6. Plant Growth Promotion Traits

Both *Burkholderia* strains displayed multiple plant growth-promoting characteristics; however, *B. vietnamiensis* was significantly more active and responsive than *B. gladioli*. The higher amylase enzymatic index, increased siderophore production, and responsive IAA synthesis in *B. vietnamiensis* have also been reported in previous literature [48,49,50]. The ability of both strains to grow in nitrogen-free LGI media demonstrates that both can fix nitrogen; this is supported by previous research indicating that *B. vietnamiensis* possesses nifH and exhibits nitrogenase activity [51].

Siderophore production, principally ornibactins, expedites efficient iron acquisition under iron-limited conditions and contributes to antifungal effects through iron competition [52,53]. IAA production, enhanced by L-tryptophan supplementation, is associated with enhanced root growth, improved nutrient uptake, and greater tolerance to abiotic stresses [54,55]. These in vitro PGP traits translated into measurable improvements in early plant development, with *B. vietnamiensis* significantly improving germination rate, root length, and shoot length at optimal concentrations (10^7^ CFU mL^−1^).

### 3.7. Biosafety Considerations

Hypersensitive response (HR) assays on tobacco and pathogenicity tests on rice clearly separated non-pathogenic *B. vietnamiensis* from pathogenic *B. gladioli* and *B. cepacia* strains that caused rapid necrosis and chlorosis. This distinction is consistent with the well-recognized dual nature of the genus *Burkholderia* [56,57]. The integrated screening approach employed here, combining HR testing, pathogenicity assays, and functional characterization, identified *B. vietnamiensis* J14EPLEAF2 as a promising low-risk biocontrol strain, while effectively rejecting high-risk isolates [47].

## 4. Materials and Methods

### 4.1. Field Design and Plant Growth

Seeds of 260 rice genotypic varieties were obtained from the Rice Research Institute, Sichuan Agricultural University. All seeds were surface-sterilized using 10% H_2_O_2_, followed by 70% ethanol, and rinsed 5 times with sterile ddH_2_O (5 min per rinse). After disinfection, seeds were germinated in sterile ddH_2_O and subsequently transferred to the field for planting at Wenjiang, Chengdu, Sichuan, China.

From these, 100 rice varieties with different sheath blight incidence rates caused by *Rhizoctonia solani* AG1IA were selected to examine the relationship between rice genotype and phyllosphere microbiome community composition (Supplementary Files S1 and S2). Selection was based on recorded disease incidence data for each variety. After four weeks, seedlings were transplanted into a paddy field in Chengdu, Sichuan Province, China. The experimental field consisted of 780 rows, with each rice variety planted in three rows.

At the tillering stage, three leaves were collected from each row per variety (nine leaves per variety). Samples were placed in ice bags and transported to the laboratory. Leaves were washed under running tap water, surface-sterilized using the same procedure described above, and stored at −20 °C until further processing.

### 4.2. Leaf Sampling and DNA Extraction

Whole leaves, including the leaf blade, ligule, auricle, and sheath, from visually healthy, asymptomatic plants were sampled at the tillering stage, which is a sensitive growth stage of rice to infection by *R. solani* AG1IA. Three bottom leaves from three different tillers of each variety were collected. Collected leaves were pooled and ground in liquid nitrogen using a sterile mortar and pestle. From each pooled sample, 50 mg of ground tissue was used for genomic DNA extraction. Three independent biological replicates were prepared for each variety.

Total genomic DNA from surface-sterilized leaf samples was extracted using the DNA Quick Plant System Kit (Tiangen Biotech, Beijing, China) according to the manufacturer’s instructions. The quantity and quality of extracted DNA were assessed using a NanoDrop spectrophotometer (Gene Company Limited, Hong Kong China) and by 1.5% agarose gel electrophoresis. DNA samples were stored in TE buffer (10 mM Tris-HCl, 1 mM EDTA, pH 8.0) at −80 °C until further analysis.

### 4.3. DNA Amplification, Sequencing and Library Preparation

The V3–V4 hypervariable regions of bacterial 16S rRNA genes were amplified using the primer set 341F (CCTAYGGGRBGCASCAG) and 806R (GGACTACNNGGGTATCTAAT) [22]. Polymerase chain reaction (PCR) was performed using 10 ng of template DNA, 0.2 µM of each primer, and 15 µL of Phusion High-Fidelity PCR Master Mix (New England Biolabs, Ipswich, MA, USA) per reaction.

The PCR cycling conditions were as follows: initial denaturation at 98 °C for 1 min, followed by 30 cycles of denaturation at 98 °C for 10 s, annealing at 50 °C for 30 s, and elongation at 72 °C for 30 s. PCR products were verified for size and purity prior to library preparation.

Sequencing libraries were prepared using the TruSeq DNA PCR-Free Sample Preparation Kit (Illumina, San Diego, CA, USA) according to the manufacturer’s instructions. Library quality and concentration were assessed using a Qubit 2.0 Fluorometer (Thermo Scientific, Carlsbad, CA, USA) and the Agilent Bioanalyzer 2100 system (Agilent Technologies, Santa Clara, CA, USA). Libraries were sequenced on the Illumina NovaSeq platform, generating 250 bp paired-end reads for downstream microbiome analysis.

### 4.4. Bioinformatic Analysis

Paired-end reads were merged using FLASH (v1.2.7) [58]. Quality control of raw tags was performed using QIIME (v1.9.1) to retain high-quality clean tags [59]. Chimera sequences were detected and removed using the UCHIME algorithm, and the resulting tags were compared against the SILVA database [60,61], yielding high-quality tags for subsequent analysis.

Sequences were clustered into operational taxonomic units (OTUs) at ≥97% similarity using Uparse software (v7.0.1001) [62]. Representative sequences from each OTU were selected for taxonomic annotation using the SILVA database based on the Mothur algorithm [61]. To investigate phylogenetic relationships among OTUs and differences in dominant taxa across groups, multiple sequence alignment was conducted using MUSCLE (v3.8.31) [63].

OTU abundance data were normalized to the sample with the fewest sequences to account for differences in sequencing depth. All downstream analyses of alpha and beta diversity were performed on these normalized data.

#### 4.4.1. Alpha Diversity

Alpha diversity metrics, including ACE, Chao1, Observed-species, Shannon, Simpson, and Good’s coverage, were calculated using QIIME (v1.7.0) and visualized in R software (v2.15.3). ACE and Chao1 were used as measures of community richness, while Shannon and Simpson indices reflected community diversity. Alpha diversity comparisons were performed between resistant and susceptible rice varieties. Sequencing depth adequacy was confirmed by rarefaction curves, which reached plateau phase for all samples, indicating sufficient coverage for downstream diversity analyses (Supplementary Figure S10).

#### 4.4.2. Beta Diversity

Beta diversity analysis was conducted to assess differences in microbial community composition between samples. Weighted and unweighted UniFrac distances and AMOVA (Analysis of Molecular Variance) were used to quantify dissimilarity between communities. Beta diversity analyses were performed using QIIME (v1.7.0) and visualized in R (v2.15.3).

Principal component analysis (PCA) was performed using the FactoMineR and ggplot2 packages in R (v4.0.2) to reduce the dataset’s dimensionality. Principal coordinate analysis (PCoA) was visualized using WGCNA, stat, and ggplot2 packages in R. Non-metric multidimensional scaling (NMDS) was also performed as a complementary method to evaluate dissimilarities among samples.

### 4.5. Isolation of Endophytic and Epiphytic Bacteria

Endophytic and epiphytic bacteria were isolated from roots, leaves, stems, and seeds of sheath-blight-resistant rice varieties grown in paddy fields in Chengdu, Sichuan Province, China, over two rice growing seasons. Bacterial isolation was performed as described by Sturz et al. [64] with minor modifications. Healthy plant tissues were collected and processed as follows: segments of roots, stems, leaves, and seeds were triturated in phosphate-buffered saline (PBS, pH 7.0). For epiphytic bacteria, 50 μL of the resulting suspension was plated onto Lysogeny Broth (LB) (Hopebio, Qingdao, China) and Nutrient Agar (NA) (Hopebio, Qingdao, China) and incubated at 25 °C and 30 °C for 48 h. For endophytic bacteria, surface-sterilized tissue segments were incubated in 250 mL Erlenmeyer flasks containing PBS (pH 7.0) with shaking at 200 rpm for 24 h at 30 °C. The resulting suspensions were streaked onto LB and NA plates and incubated at 25 °C and 30 °C for 48 h.

### 4.6. Molecular Identification and Phylogenetic Analysis

Bacterial isolates demonstrating antagonistic activity against *Rhizoctonia solani* AG1IA were selected for genomic DNA extraction using the Bacterial Genomic DNA Extraction Kit (Sangon Biotech, Shanghai, China) according to the manufacturer’s instructions.

The 16S rRNA gene was amplified using universal primers 27F (5′-AGAGTTTGATCCTGGCTCAG-3′) and 1492R (5′-GGTTACCTTGTTACGACTT-3′). PCR was performed on a BIO-RAD T100 Thermal Cycler under the following conditions: initial denaturation at 94 °C for 4 min, followed by 35 cycles of denaturation at 94 °C, annealing at 60 °C for 1 min, and extension at 72 °C for 2 min. PCR products were sequenced, and resulting sequences were aligned using BLAST (v2.16.0) against the NCBI database for taxonomic identification [65].

Multiple alignments of the resulting sequences were performed using CLUSTALW, and phylogenetic trees were created by maximum-likelihood methods in MEGA software (Version 7.0), with bootstrap resampling (1000 iterations) to support the tree topology.

### 4.7. In Vitro Antagonistic Assay Against R. solani AG1IA

All isolated bacterial strains were tested for their potential to suppress the mycelial growth of *Rhizoctonia solani* AG1IA using a dual-culture assay [66]. Bacterial isolates were grown on Lysogeny Broth (LB) agar for 48 h, and a single loopful of active bacterial culture was streaked at the centre of a 9 cm Petri dish. Mycelial discs (5 mm in diameter) of *R. solani* AG1IA were obtained from 3-day-old cultures and inoculated onto Potato Dextrose Agar (PDA) plates. Plates were incubated at 25 °C for four days, and fungal growth was monitored daily. The inhibition zone between bacterial colonies and fungal mycelium was measured. Three plates were used per bacterial isolate, and the experiment was repeated independently four times to ensure reproducibility.

Radial growth of *R. solani* AG1IA (D1, in mm) in dual culture was recorded, and the growth inhibition percentage (GIP) was calculated using the formula [67]:

$$GIP\left(\%\right)=\frac{D_{2}-D_{1}}{D_{2}}\;\times \;100$$

where D1 is the radial growth of *R. solani* AG1IA in the presence of bacterial isolate, and D2 is the radial growth of *R. solani* AG1IA in the control plate without bacterial inoculation.

### 4.8. Extraction of Intracellular Metabolite

Bacterial cells were collected by centrifugation at 10,000 rpm, 4 °C, for 20 min after 3 days of growth in LB at 37 °C and 170 rpm. After washing twice, the cells were suspended in PBS buffer and placed in sonication tubes. The samples were then sonicated for 20 min and centrifuged (10,000 rpm, 4 °C, and 20 min).

### 4.9. Detached Leaf, Greenhouse and Field Bioassays

#### 4.9.1. Detached Leaf Bioassay and Greenhouse Bioassay

To evaluate the inhibitory effect of isolated *Burkholderia* spp. on *Rhizoctonia solani* AG1IA infection, detached leaf assays were performed. The fourth leaf from the base of a three-month-old susceptible rice variety (Lemont) was collected. Leaves were cut into 15 cm segments, surface-sterilized with 1% sodium hypochlorite, rinsed with sterile double-distilled water (ddH_2_O), and placed on trays containing wet filter paper.

*Burkholderia* spp. were cultured overnight in Lysogeny Broth (LB) at 30 °C with shaking (200 rpm). Cells were harvested by centrifugation (5000 rpm, 10 min), washed twice with sterile ddH_2_O, and resuspended to a concentration of 10^6^ CFU/mL. Tween 20 (10 μL) was added, and the bacterial suspension was evenly spread over the leaf surface using a sterile cotton swab. A 5 mm mycelial plug of actively growing *R. solani* AG1IA was placed in the center of each leaf segment.

Leaves treated with sterile ddH_2_O, rather than bacteria, served as negative controls. Leaf segments were incubated under laboratory conditions for ten days, and sclerotia formation and lesion development were recorded [34].

To assess the effect of isolated endophytic bacteria under controlled conditions, surface-sterilized rice seeds were germinated in sterile water and transferred to pots containing sterilized soil. Soil was treated with 10^6^ CFU of each *Burkholderia* species. One-month-old seedlings were inoculated with *R. solani* AG1IA using toothpicks containing actively growing mycelium. Disease progression and symptom development were monitored over time.

#### 4.9.2. Field Bioassay

To evaluate the influence of endophytic bacteria under natural conditions, three-month-old rice plants were artificially inoculated with *Burkholderia* suspensions (10^6^ CFU/mL) and then inoculated with *R. solani* AG1IA. Disease development and symptom progression were recorded to assess biocontrol efficacy under field conditions. All experiments were conducted with independent biological replicates to ensure reproducibility.

### 4.10. Observation of R. solani Infection Structures

The formation of infection cushions and symptom development by *Rhizoctonia solani* AG1IA in the presence or absence of *Burkholderia* species were monitored daily for seven days post inoculation. Fungal structures in rice leaves were stained using the method of Nassimi and Taheri [35]. Detached leaf pieces were fixed in acetic acid–ethanol solution (63% ethanol, 5% acetic acid) and subsequently cleared in lactophenol at 37 °C. Fungal infection structures were stained with Trypan blue in lactophenol and examined under a ZEISS AX10 light microscope (Jena, Germany). For each *Burkholderia* species, three biological replicates were evaluated, and the experiment was independently repeated three times to ensure reproducibility.

### 4.11. Hypersensitive Response (HR) Assay and Pathogenicity Evaluation

To determine the ability of *Burkholderia* species to induce a hypersensitive response (HR), 50 µL of bacterial suspension (10^7^ CFU mL^−1^) was infiltrated into fully expanded leaves of six-week-old tobacco (*Nicotiana benthamiana*) plants. As a negative control, 50 µL of sterile ddH_2_O was infiltrated into leaves. Each treatment was applied to four independent leaves, and the experiment was repeated three times [68].

Pathogenicity assays on rice were performed as described by Schaad et al. [69] with minor modifications. Leaf blades, sheathes, and panicles of four-month-old Lemont rice plants were clipped and sprayed with bacterial suspensions (10^7^ cfu/mL). Plants were then covered with parafilm and grown in a greenhouse at temperatures of 35–37 °C, 75% relative humidity, and 16 h of light per 24 h cycle. Sterile ddH_2_O was sprayed onto control plants. Each treatment was repeated three times.

Symptoms in treated plants were monitored daily, and chlorosis and water-soaked lesions were evident on all leaves (blade, sheath, panicle) of plants sprayed with pathogenic bacterial suspensions within 3 days. Plants treated with non-pathogenic bacterial suspensions (*B. vietnamiensis*) did not show symptoms, whereas controls showed none. The pathogen could be reisolated from symptomatic rice tissue to fulfill Koch’s postulates.

### 4.12. RNA Extraction and Quantitative Real-Time PCR (qPCR)

Total RNA was extracted from 50 mg of rice leaf tissue using TRIzol reagent (Thermo Fisher Scientific, Waltham, MA, USA) according to the manufacturer’s instructions. To remove contaminating genomic DNA, the samples were treated with RNase-free DNase I (Promega, Madison, WI, USA). RNA quality and concentration were assessed using a NanoDrop spectrophotometer (Thermo Fisher Scientific, Waltham, MA, USA).

First-strand cDNA was synthesized using a reverse transcription kit (Thermo Fisher Scientific, Waltham, MA, USA) according to the manufacturer’s instructions. Quantitative real-time PCR (qPCR) was carried out on a StepOnePlus Real-Time PCR machine (Applied Biosystems, Waltham, MA, USA) using the QuantiTect SYBR Green RT-PCR kit (Qiagen, Germantown, MD, USA). The qPCR conditions were: denaturation at 95 °C for 15 s, amplification at 60 °C for 30 s, and extension at 72 °C for 30 s, repeated for 40 cycles. Cycle threshold values were used to calculate the relative expression of defense-related genes involved in the salicylic acid (SA) and jasmonic acid (JA) signaling pathways using the 2^−ΔΔCt^ method [70].

### 4.13. Plant Growth-Promoting (PGP) Trait Assays

#### 4.13.1. Seedling Growth Promotion

Rice seeds (*Oryza sativa* L. cv. Zhonghua 11) were surface-sterilized by immersion in 70% ethanol for 1 min, followed by 2% sodium hypochlorite for 3 min, and then rinsed five times with sterile ddH_2_O. Sterilized seeds were soaked for 2 h in suspensions of *Burkholderia vietnamiensis* or *Burkholderia gladioli* adjusted to 10^5^, 10^6^, 10^7^, 10^8^, or 10^9^ CFU mL^−1^; seeds treated with sterile ddH_2_O served as mock controls.

The treated seeds were placed on sterile, damp filter paper within Petri dishes and incubated (in the absence of light) at 28 °C for 72 h to assess seed germination. The rate of seed germination was then calculated as a percent of seeds showing a visible radicle. After germination, root length was measured by utilizing a digital caliper. Three independent biological replicates per treatment, each containing at least 10 seeds, were used to determine the average root length for each treatment.

#### 4.13.2. Siderophore Production Assay

Siderophore production by *Burkholderia* isolates was evaluated using the Chrome Azurol S (CAS) agar plate assay as described by Schwyn and Neilands [71] with minor modifications. Bacterial strains were initially grown overnight in LB at 30 °C with shaking at 180 rpm. Cultures were adjusted to an optical density (OD_600_) of 0.5 using sterile saline. CAS agar medium was prepared by combining the CAS indicator solution with iron-deficient minimal medium, autoclaving each separately, and mixing under sterile conditions. Aliquots (5 µL) of standardized bacterial suspensions were spot-inoculated onto CAS agar plates. Plates were incubated at 30 °C for 48–72 h.

Siderophore production was indicated by the formation of an orange or yellow halo surrounding bacterial colonies against the blue background of the CAS medium. Siderophore production in liquid culture was quantified using the CAS shuttle assay. Cell-free supernatants were obtained by centrifugation of 5 mL cultures at 10,000× *g* for 10 min. Equal volumes (0.5 mL) of supernatant and CAS reagent were mixed and incubated in the dark for 20 min. Absorbance was measured at 630 nm using a spectrophotometer. Percentage siderophore units were calculated as:

$$\%\;Siderophore=\frac{A_{r}-A_{s}}{A_{r}}\;\times \;100$$

where A_r_ is the absorbance of the reference (CAS reagent + uninoculated media), and A_s_ is the absorbance of the sample (CAS + culture supernatant).

#### 4.13.3. Indole-3-Acetic Acid (IAA) Production Assay

Indole-3-acetic acid (IAA) production by *B. gladioli* and *B. vietnamiensis* was assessed in King’s B (KB) broth. Bacterial cultures were grown in KB at 28 °C with shaking at 150 rpm for 48 h. For L-tryptophan-amended treatments, KB was supplemented with 0.5 g L^−1^ L-tryptophan before inoculation, while uninoculated KB served as the medium control.

The IAA levels were measured using the Salkowski colorimetric method [72] after incubation. The culture supernatants were then centrifuged at 10,000× *g* for 10 min to remove the cells. One millilitre of supernatant was then combined with 2 mL of Salkowski reagent (1 mL of 0.5 M FeCl_3_ in 50 mL of 35% perchloric acid) and incubated for 30 min at room temperature in the dark. A pink colour development confirmed that the bacteria had produced IAA; absorbance was measured at 530 nm, and IAA concentrations were determined by reference to a calibration curve constructed with various concentrations of commercially available IAA.

#### 4.13.4. Amylase Activity

The starch agar plates were made by adding 1% (*w*/*v*) soluble starch to nutrient agar, autoclaving at 121 °C for 15 min to sterilize the media, and then pouring into sterile Petri dishes under aseptic conditions. Once the agar had solidified, the bacteria were plated onto the plates using a “spot” inoculation technique, and the plates were incubated at 28 °C for 48 h, with the uninoculated plates serving as the control.

Following incubation, plates were flooded with iodine solution and allowed to react for 2–3 min; excess iodine was then gently decanted, and plates were examined for clear zones surrounding colonies. The presence of a clear halo against the dark, iodine-stained background indicated starch hydrolysis and extracellular amylase activity.

Amylase activity was expressed as an enzymatic index (EI). The diameters of the clear zone and the corresponding bacterial colony were measured using a digital calliper, and EI was calculated as the ratio of clear-zone diameter to colony diameter:

$$Enzymatic\;Index\left(EI\right)=\frac{Diameter\;of\;hydrolysis\;zone}{Diameter\;of\;Colony}$$

#### 4.13.5. Phosphate Solubilization Assay

Phosphate solubilization by *B. vietnamiensis* J14EPLEAF2 and *B. gladioli* A1EPSEED5 was evaluated using National Botanical Research Institute’s Phosphate (NBRIP)( Hopebio, Qingdao, China) broth supplemented with bromophenol blue (BPB) as a pH indicator [73]. Uninoculated BPB–NBRIP medium served as the negative control. Bacterial strains were grown overnight in LB at 28 °C with shaking. Cultures were adjusted to a uniform cell density and inoculated into BPB–NBRIP broth containing tricalcium phosphate as the sole insoluble phosphorus source. Inoculated cultures were incubated at 28 °C with shaking for up to 120 h. Phosphate solubilization activity was monitored at 72, 96, and 120 h post inoculation by measuring absorbance at 595 nm (OD_595_) using a spectrophotometer. The pH of the culture medium was measured concurrently at each time point using a calibrated digital pH meter.

#### 4.13.6. Proteolytic Activity Assay

Proteolytic activity of selected *Burkholderia* isolates was evaluated using a skim milk agar assay to detect extracellular protease production. Skim milk agar medium was prepared by supplementing nutrient agar with skim milk powder (1–2%, *w*/*v*). The medium was autoclaved and poured into sterile Petri dishes under aseptic conditions. Bacterial isolates were grown overnight in LB at 28 °C with shaking, then adjusted to an optical density (OD_600_) of 0.5. Aliquots (5 µL) of standardized cultures were spot-inoculated onto the surface of skim milk agar plates. Plates were incubated at 28 °C for 48–72 h.

Proteolytic activity was indicated by the formation of clear or translucent halo zones surrounding bacterial colonies due to casein hydrolysis. Halo and colony diameters were measured using a digital caliper, and proteolytic activity was expressed as a proteolytic index (PI), calculated as:

$$PI=\frac{Diameter\;of\;clear\;zone}{Diameter\;of\;colony}$$

### 4.14. LC-MS Analysis

#### 4.14.1. Sample Preparation and Extraction

A centrifuge tube was used to extract 0.5 to 1.0 mL of liquid sample. An extraction solvent of methanol/acetonitrile (1:1) was added to the sample, with a volume 2× that of the sample (1.0–2.0 mL). The sample was then mixed with the extraction solvent for 60 s. The mixed sample was then subjected to ultrasonic extraction at 4 °C for 30 min to extract the metabolites. The sample was then centrifuged at 12,000 rpm for 10 min at 4 °C to separate the solid and liquid phases. The supernatant was then placed at −20 °C for 1 h to allow any proteins to precipitate out. The sample was then centrifuged again at 12,000 rpm for 10 min at 4 °C to remove any remaining precipitate. The supernatant was transferred to a new centrifuge tube. The sample was then dried using a vacuum dryer to remove any solvents. To prepare the sample for further analysis, the dried residue was dissolved in 100 μL of a 30% acetonitrile solution.

#### 4.14.2. UPLC Analysis

The sample extract was analyzed by a high-resolution UPLC-Orbitrap-MS system (UPLC, Vanquish; MS, HFX). The liquid chromatography conditions used for analysis were as follows: The column used was a Waters HSS T3 column (100 × 2.1 mm, 1.8 μm). The column temperature was set to 40 °C, and the flow rate was 0.3 mL/min. The injected volume was 2 μL. The solvent system consisted of water (0.1% acetic acid) and acetonitrile (0.1% acetic acid). The gradient program was: 0 min: 100% A/0% B; 1 min: 100% A/0% B; 4 min: 40% A/60% B; 6.5 min: 5% A/95% B; 6.6 min: 100% A/0% B; 8.0 min: stop.

#### 4.14.3. MS Conditions

Mass spectrometry data were obtained using a Q Exactive HFX Hybrid Quadrupole-Orbitrap mass spectrometer (Thermo Fisher Scientific, Waltham, MA, USA) equipped with a heated electrospray ionization (ESI) source. The data were acquired in Full-ms-ddMS2/MS modes. The ESI source parameters were: spray voltage: −2.8 kV (negative)/+3.0 kV (positive); sheath gas pressure: 40 arb; auxiliary gas pressure: 10 arb; sweep gas pressure: 0 arb; capillary temperature: 320 °C; auxiliary gas heater temperature: 350 °C.

#### 4.14.4. Data Processing and Analysis

The raw data were processed using Xcalibur software (v4.2) (Thermo Fisher Scientific). The resulting chromatograms were subjected to peak identification, alignment, and quantification. The *m*/*z* values and retention times of the detected metabolites were compared against databases such as HMDB, KEGG, and PubChem for compound identification.

### 4.15. Statistical Analysis

All experiments were performed with at least three independent biological replicates. Data are presented as mean ± standard error (SE). Statistical significance was determined using Student’s *t*-test or one-way ANOVA, followed by Duncan’s multiple range test. Differences were considered significant at *p* < 0.05 (*), *p* < 0.01 (**), or *p* < 0.001 (***). Alpha diversity comparisons were performed using Wilcoxon rank-sum tests. Beta diversity significance was assessed using PERMANOVA and AMOVA.

## 5. Conclusions

This study demonstrates that sheath blight resistance in rice is associated with distinct, diversity-rich phyllosphere microbiomes enriched in beneficial *Burkholderiaceae*, *Bacillaceae*, and *Pseudomonadaceae*. *Burkholderia vietnamiensis* J14EPLEAF2 is a promising, safe biocontrol agent with potent antifungal activity (77% inhibition), broad-spectrum antagonism, multiple PGP traits, and defense priming capacity. The strain effectively suppresses disease under laboratory, greenhouse, and field conditions by reducing lesion development, sclerotia formation, and infection cushion formation. Biosafety assessment confirms that *B. vietnamiensis* is nonpathogenic to rice and tobacco, supporting its potential for commercial bioinoculant development. These findings provide a foundation for microbiome-assisted breeding and evidence-based selection of safe, effective biocontrol agents for sustainable rice production.

## Figures and Tables

**Table 1:** Alpha Diversity Comparison Among Resistance Groups (ACE Index).

	Difference	*p* Value	Sig.	LCL	UCL
group1–group2	44.33733734	5.00 × 10^−4^	***	19.41759929	69.25707539
group1–group3	10.14414414	0.6202		−50.39315205	30.10486376
group2–group3	54.48148148	0.0171	*	−99.18023523	−9.782727735

Values represent median differences in the ACE index between groups. group1: highly resistant rice varieties, group 2: tolerant rice varieties, group 3: susceptible rice varieties. LCL: Lower Confidence Limits, UCL: Upper Confidence Limits. p < 0.001 *, * represent significance, p < 0.01. *** represents significance.

**Table 2:** Richness and Diversity analysis.

Group	Observed Species	Shannon	Simpson	Chao1	ACE	Goods Coverage	PD Whole Tree
group1	94	1.087	0.412	109.571	113.861	1.000	30.193
group2	55	1.038	0.416	64.074	66.551	1.000	24.485
group3	78	1.046	0.400	91.087	92.900	1.000	34.136

*Group 1: high-resistance rice varieties, group 2: mid resistance rice varieties, group 3: susceptible rice varieties.*

**Table 3:** Analysis of molecular variance (AMOVA) of phyllosphere bacterial communities among rice resistance groups based on unweighted UniFrac distances.

Vs-Group	ss	df	MS	FS	*p*-Value
group1–group3	0.423103 (66.032)	1 (238)	0.423103 (0.277445)	1.52499	0.049
group1–group2	0.426775 (75.4369)	1 (274)	0.426775 (0.275317)	1.55012	0.043
group2–group3	0.485696 (18.2808)	1 (70)	0.485696 (0.261155)	1.8598	0.015 *
group1–group2–group3	0.864982 (79.8748)	2 (291)	0.432491 (0.274484)	1.57565	0.01 *

* represents significance.

## Data Availability

The original contributions presented in this study are included in the article/Supplementary Material. Further inquiries can be directed to the corresponding author.

## Supplementary Materials

The following supporting information can be downloaded at: https://www.mdpi.com/article/10.3390/ijms27114879/s1.
