# Supplementary material for: Rice Genotype-Dependent Phyllosphere Microbiome Assembly and Isolation of Antagonistic Burkholderia for Sheath Blight Biocontrol
Source: Int J Mol Sci. 2026 May 28;27(11):4879. doi: 10.3390/ijms27114879 (PMC13256689; doi:10.3390/ijms27114879)
Supplement: Supplementary file 1 [file ijms-27-04879-s001.zip › ijms-4216598-Supplementary Materials figures.pdf]

Supplementary Materials

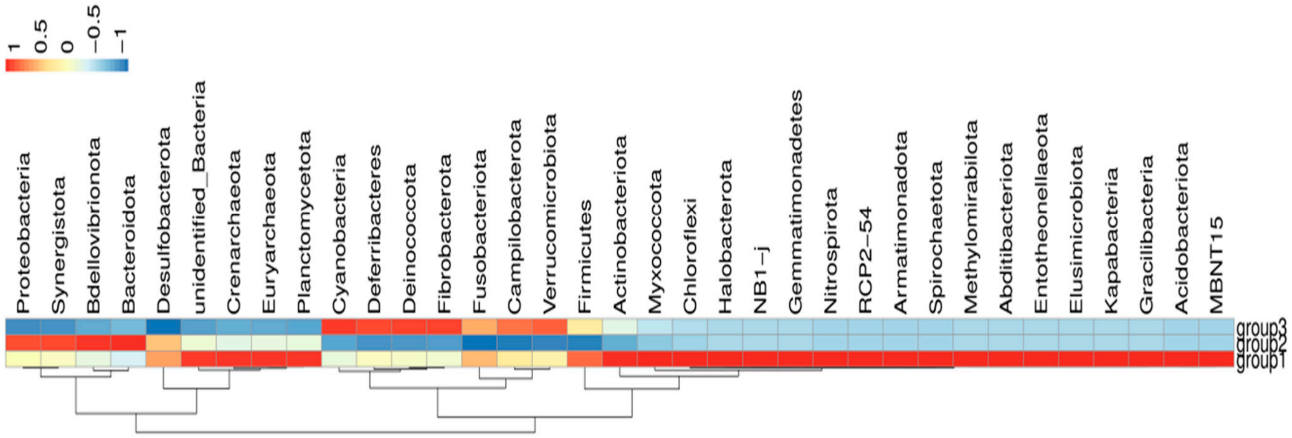

Supplementary Figure S1: OTU analysis-taxa heat map showing clustering of microbial phyla across resistance groups. (Group 1: High Resistance Rice Varieties; Group 2: Mid Resistance Rice Varieties; Group 3: Susceptible Rice Varieties)

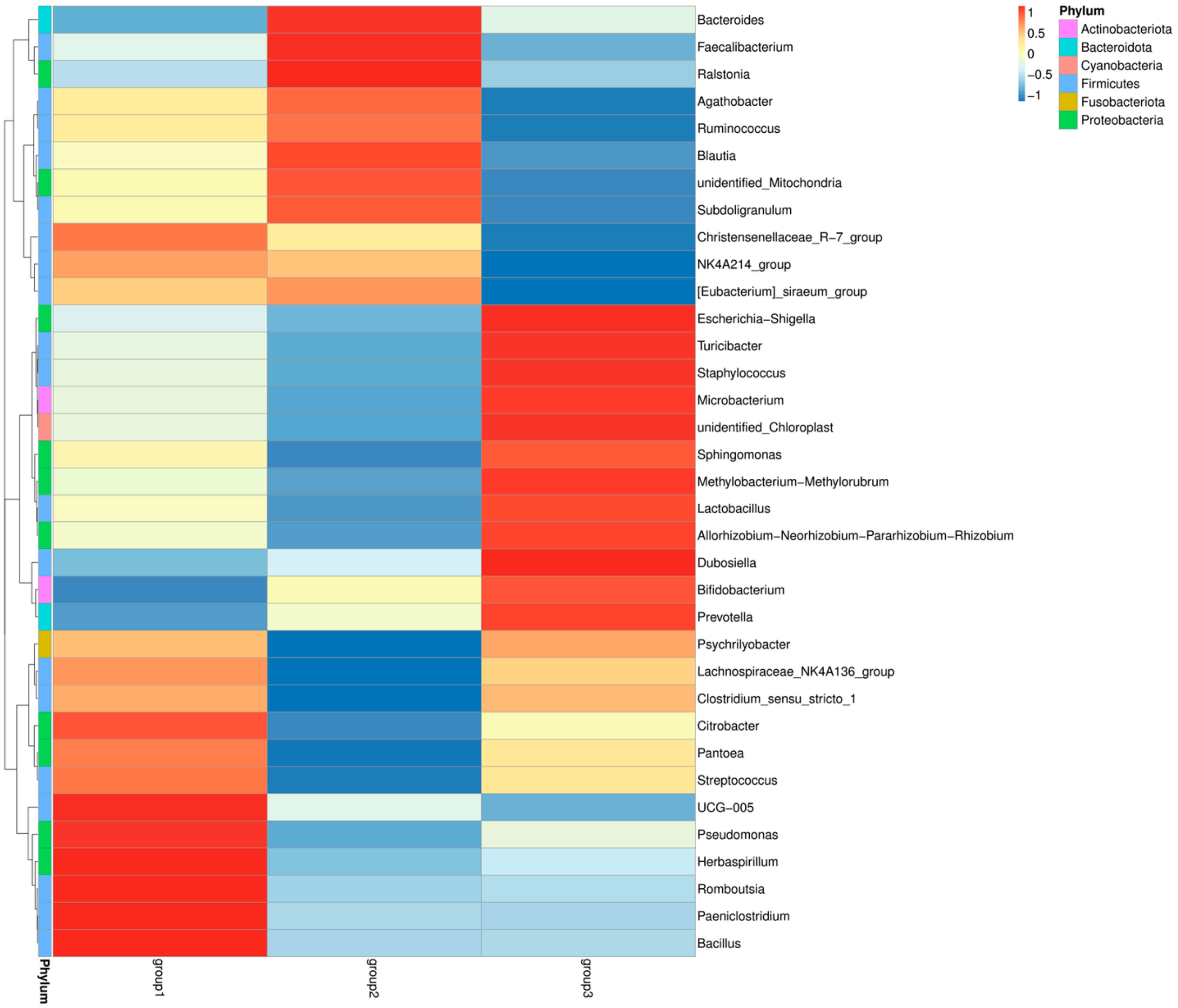

Supplementary Figure S2: Genus-level taxonomic composition of the rice phyllosphere across resistance groups. The figure illustrates the relative abundance of key bacterial genera in resistant, moderately resistant, and susceptible rice varieties. Bacillus, Pseudomonas, Paeniclostridium, and Ralstonia were more abundant in resistant and moderately resistant varieties, while Lactobacillus and Methylobacterium were prevalent in susceptible cultivars. The size of each colored segment corresponds to the proportional abundance of the genus within each resistance category (red: high resistant; blue: tolerant/moderately resistant; green: susceptible)

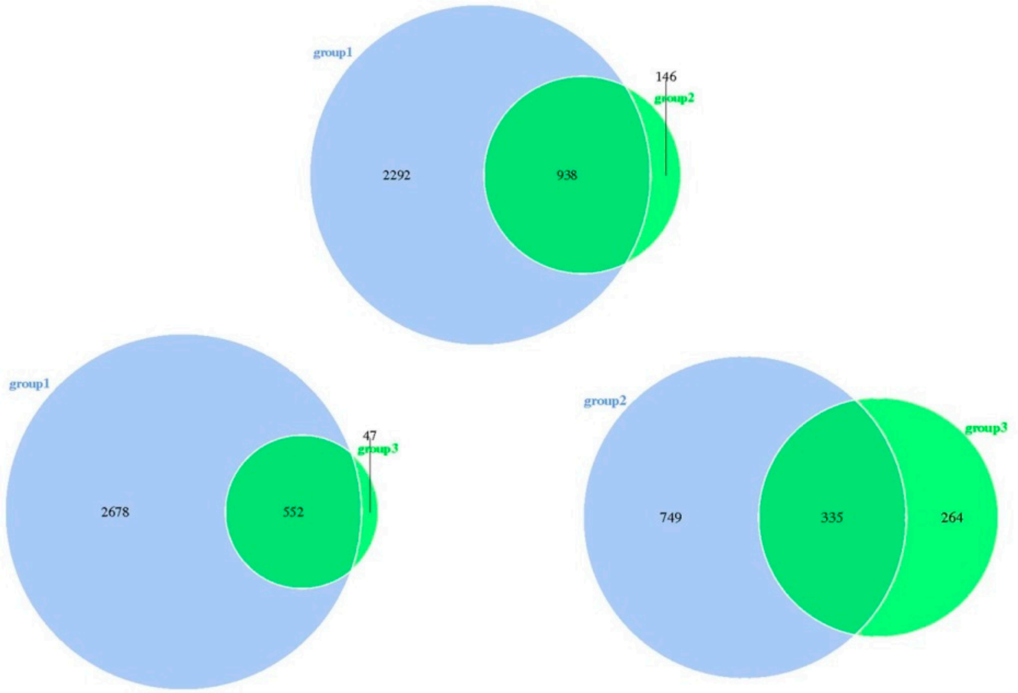

Supplementary Figure S3: Venn diagram of microbial communities with unique and shared operational taxonomic units) among three groups of rice varieties. The numbers in the Venn diagram indicate the number of OTUs in each group. group1: high resistance rice varieties, group 2: mid resistance rice varieties, group 3: susceptible rice varieties.

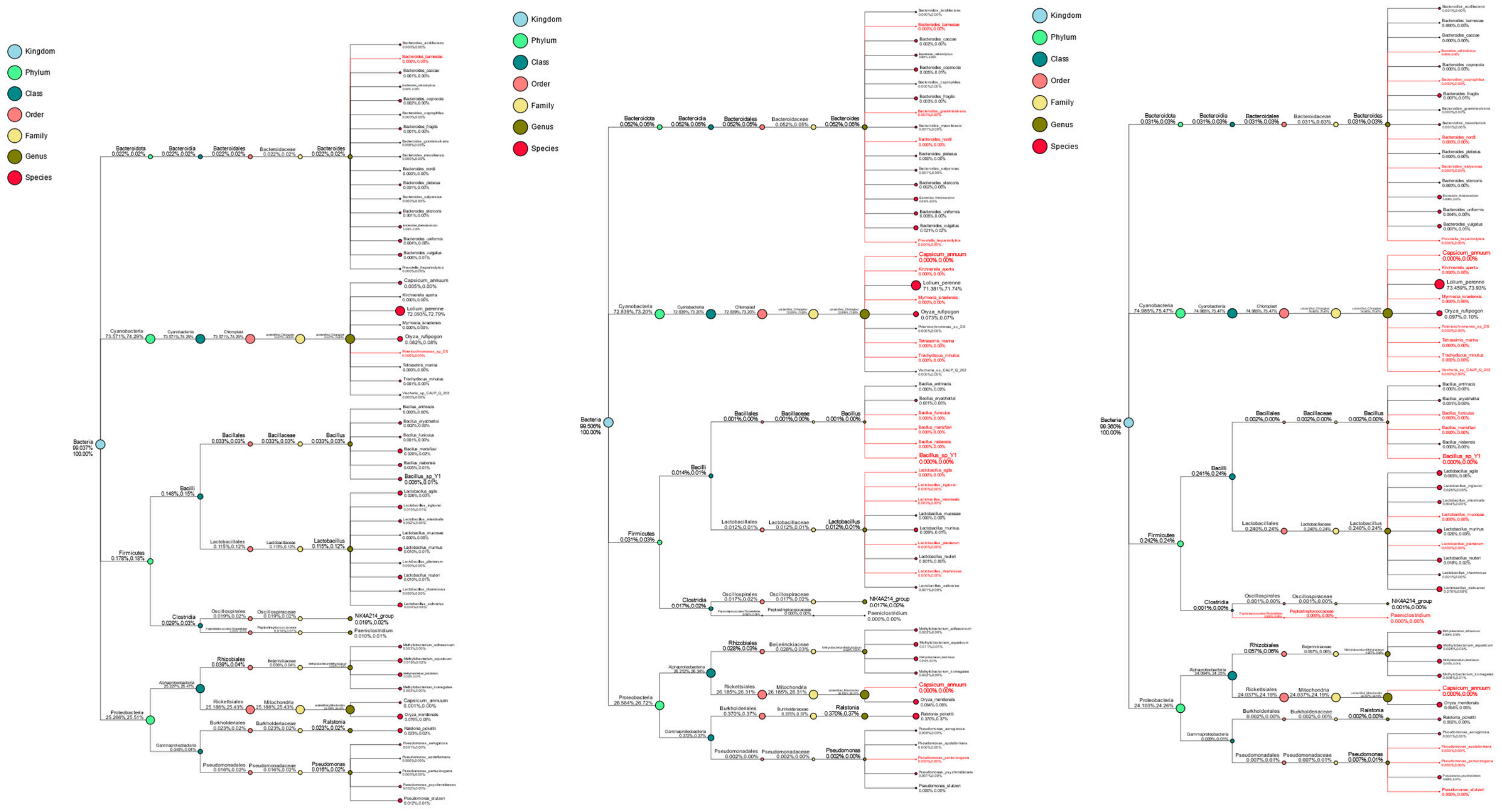

(**a**) (**b**) (**c**)

Supplementary Figure S4: UPGMA clustering tree at species level for microbial communities in (a) High Resistance Rice Varieties (b) Mid-resistance rice varieties (c) Susceptible Rice varieties

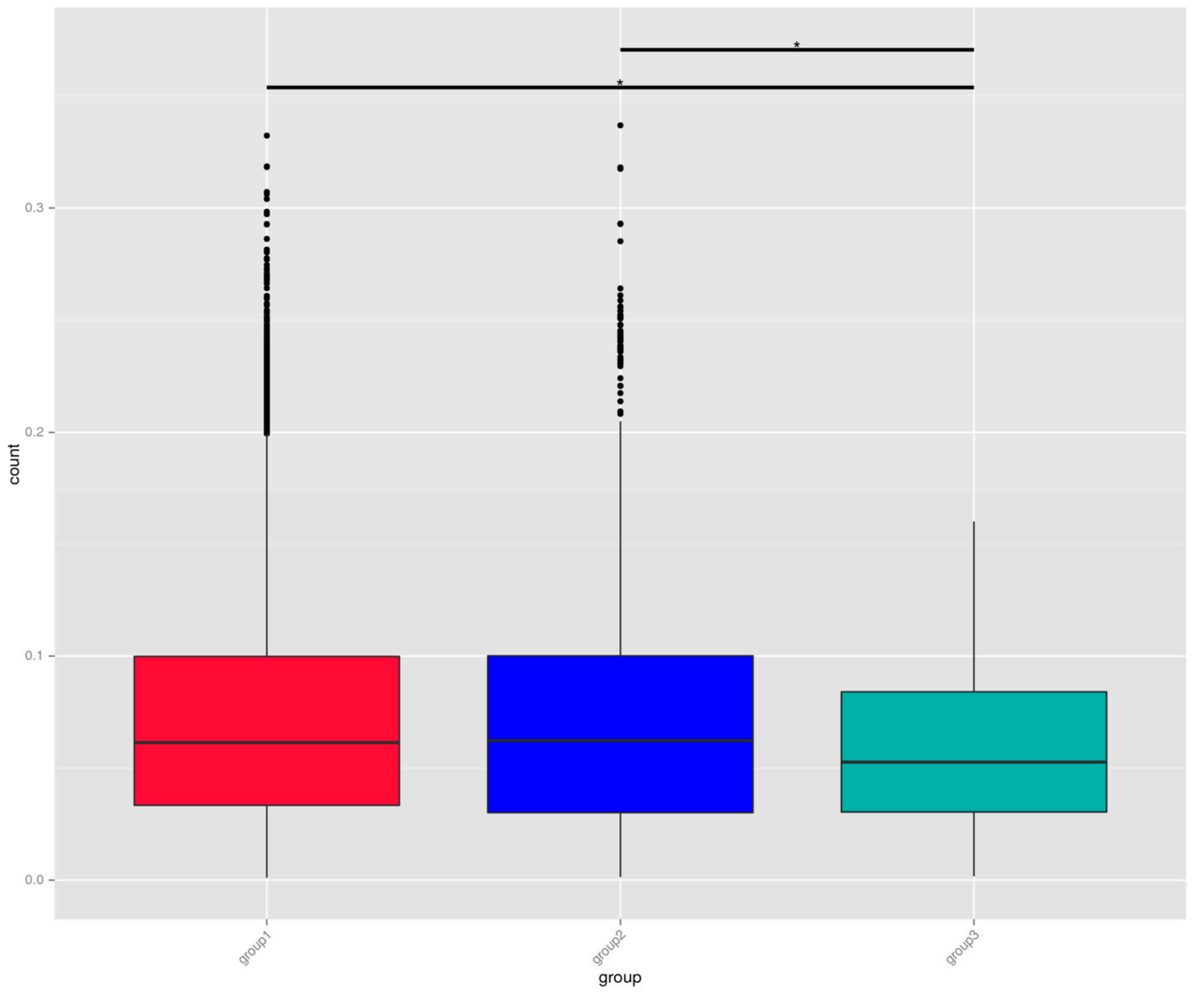

Supplementary Figure S5: Weighted UniFrac distance analysis of phyllosphere bacterial communities. The boxplot shows the distribution of weighted UniFrac distances among resistant (group 1), moderately resistant (group 2), and susceptible (group 3) rice varieties. This analysis, which accounts for relative abundance of taxa, confirms the community differentiation patterns observed with unweighted UniFrac distances.

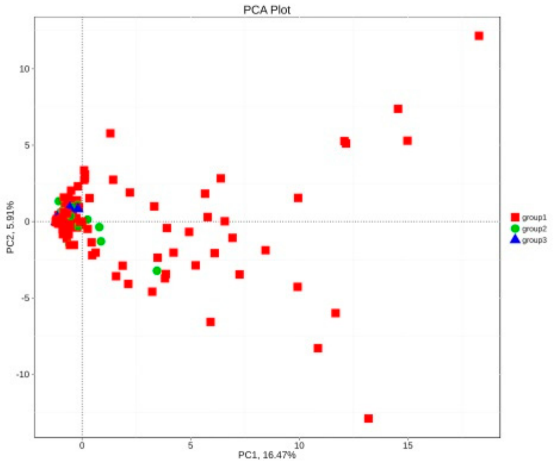

(**a**)

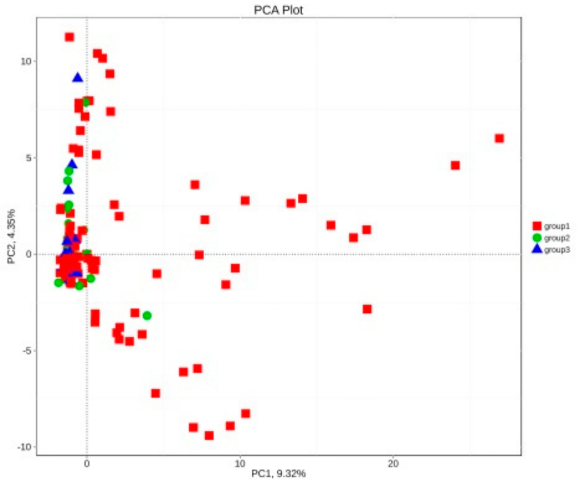

(**b**)

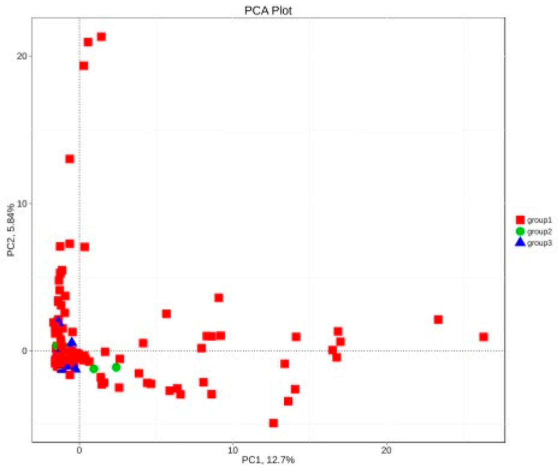

(**c**)

Supplementary Figure S6: Metastat analysis of microbial community in rice varieties at the different taxonomic levels. a) family, PC1 is the principal component causing the largest difference in samples, with an explanatory value of 9.32%. PC2 was next, with an explanatory value of 4.35%. b) Class, PC1 is the principle component causing the largest difference in samples, with an explanatory value of 16.47%. PC2 was next, with an explanatory value of 5.91%. c) Order, PC1 is the principle component causing the largest difference in samples, with an explanatory value of 12.7%. PC2 was next, with an explanatory value of 5.84%. group1: high resistance rice varieties, group 2: mid resistance rice varieties, group 3: susceptible rice varieties.

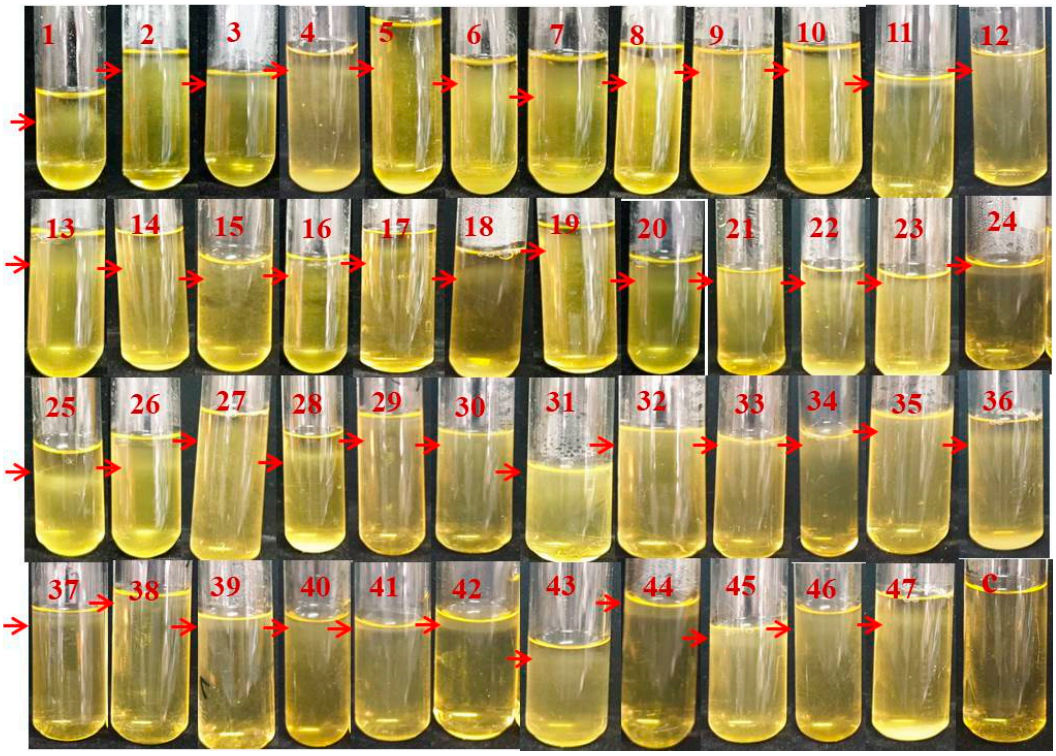

Supplementary Figure S7: Nitrogen-fixing bacteria by monitoring the pellicle formation in LGI media. 1 to 47 bacteria with nitrogen-fixing ability, and C is the control.

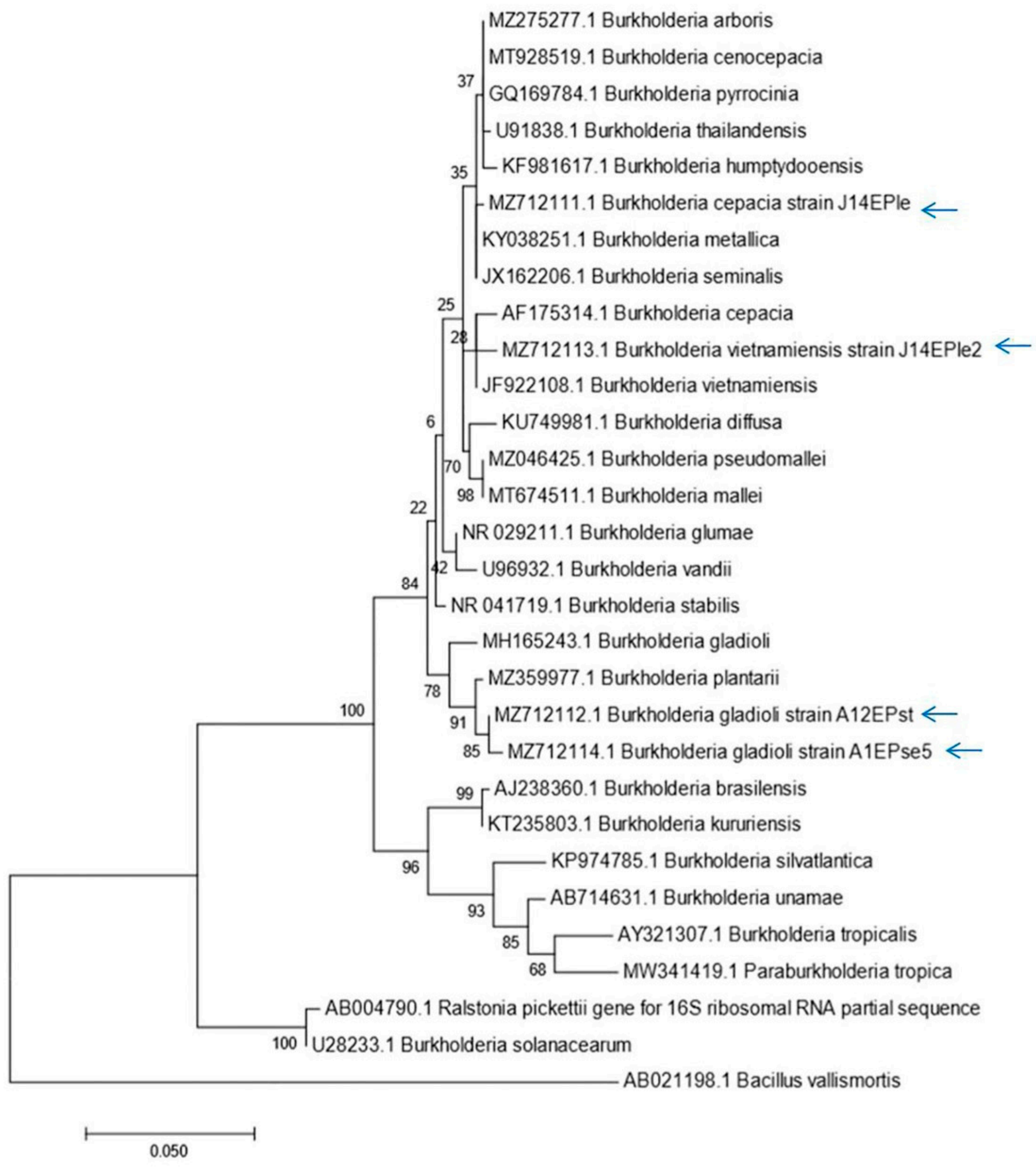

Supplementary Figure S8: Molecular phylogenetic analysis of *Burkholderia* species based on 16S rRNA. Sequences were aligned with the CLUSTALW algorithm, and the phylogenetic trees were constructed using the Maximum likelihood method (bootstrap1000) with MEGA (Version 7.0). Arrow represents four isolated *Burkholderia* spp. with inhibitory effect against *Rhizoctonia solani* AG1IA. Along each branch, the percentage of trees in which the associated taxa clustered together is shown

A

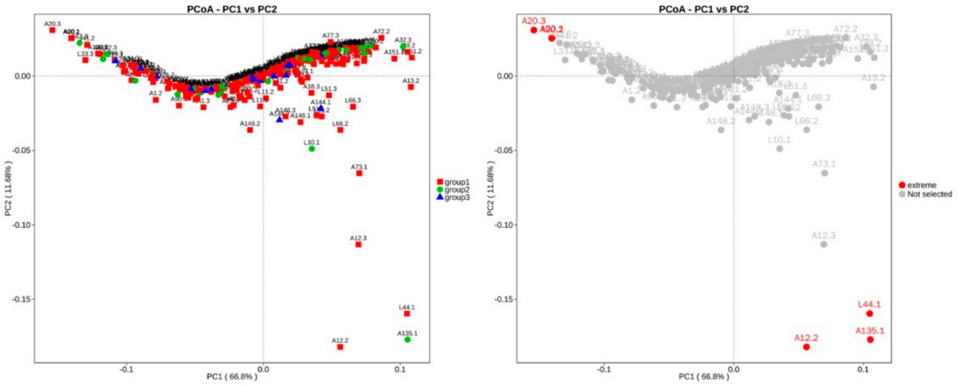

B

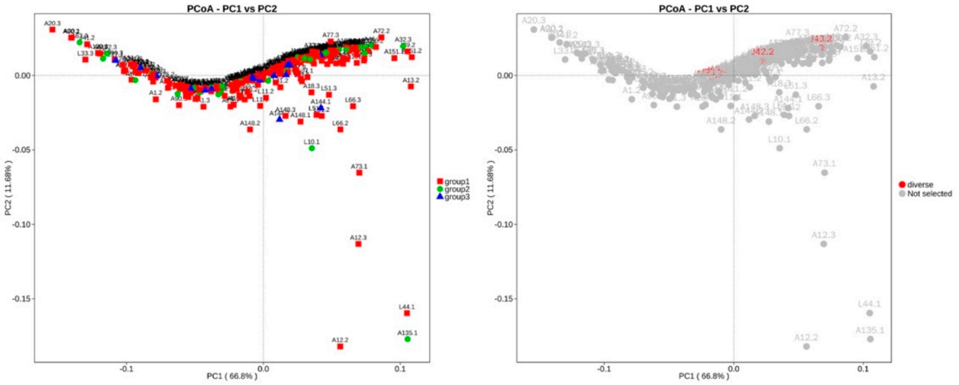

C

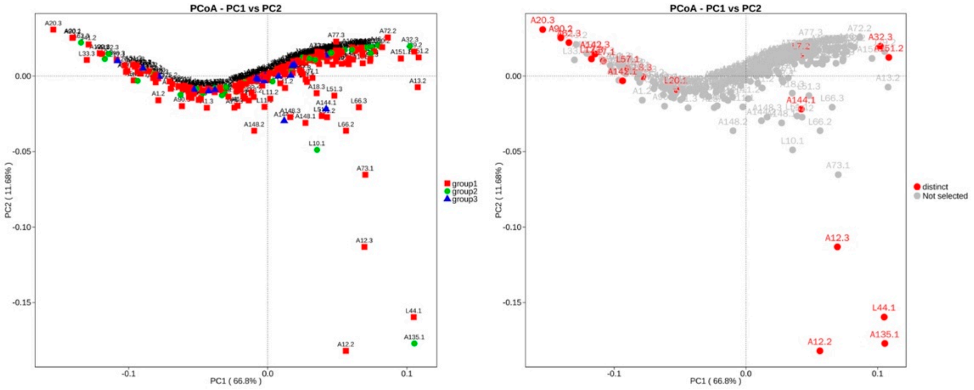

Supplementary Figure S9: OTU analysis by microPITA for select taxonomic abundance. a) Rice varieties with most dissimilarity in microbial community. b) Rice varieties with maximum diversity in the microbial community. c) distinct microbial community. PC1 is the principal coordinate component causing the largest difference in samples, with an explanatory value of 66.8%. PC2 was next, with an explanatory value of 11.69%. group1: high resistance rice varieties, group 2: mid resistance rice varieties, group 3: susceptible rice varieties. A20: CR 16-3, A135: IR10A115, A90: CT 15765-18-1-5-3-2-M, L44: Xiangwanxian3hao, A12:

Bawangbian1, A33: RP 2071-22-4-1R, J43: 84R, J13: Zenggongliaogeng, J33: R715, J42: 48R, A90: CT 15765-18-1-5-3-2-M, A151: CT 15696-3-4-1-1-1-M, A12: P 1356-1-3M-2-1B, L44: Xiangwanxian3hao, L7: Xiangaizao10, A142: IR 09A101, L20: Laozaogu, L8: Jinnante43B, A144, L57: Lemont, L17: JinnanteB.

A B

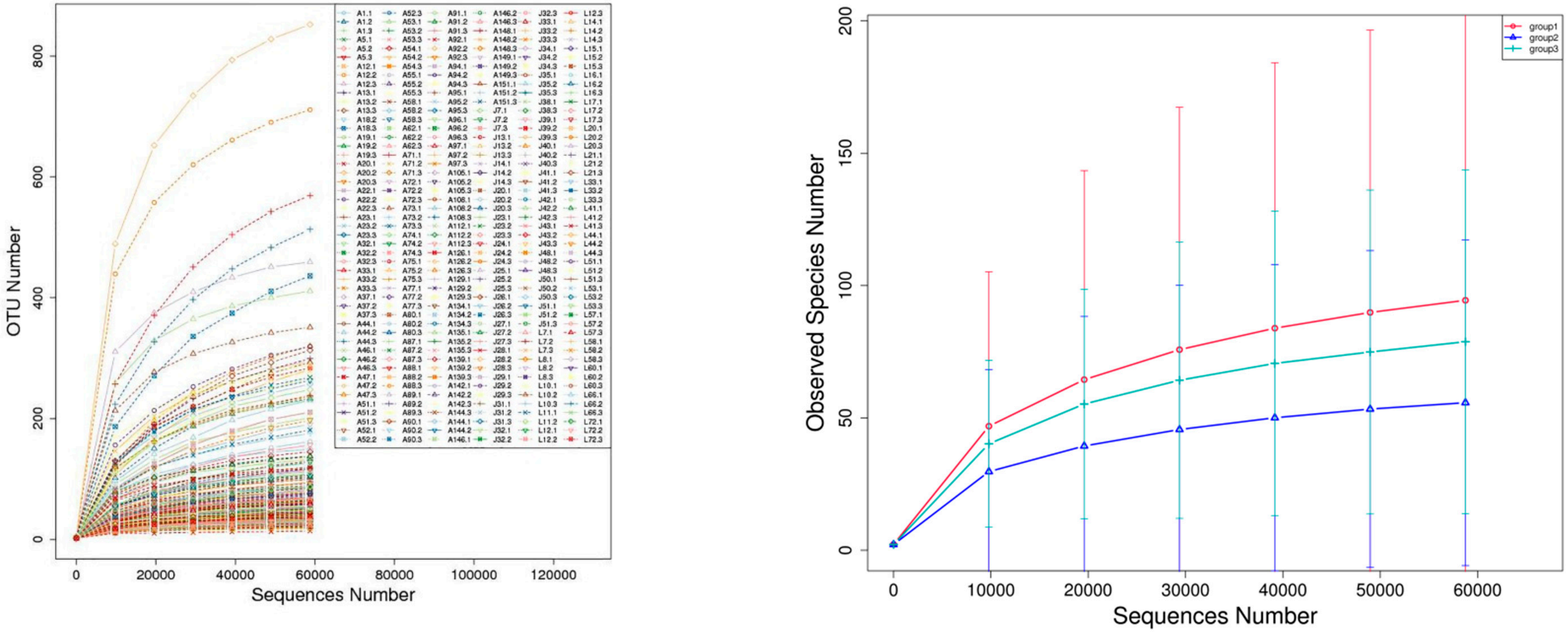

Supplementary Figure S10 Structure of the Phyllosphere microbiotas based on 16S rDNA- amplicon pyrosequencing. (a) Rarefaction curves of operational taxonomic units (OTUs). (b) Observed species number (60,000 sequences) in the phyllosphere of group 1, group 2, and group 3 rice varieties at the tillering stage.

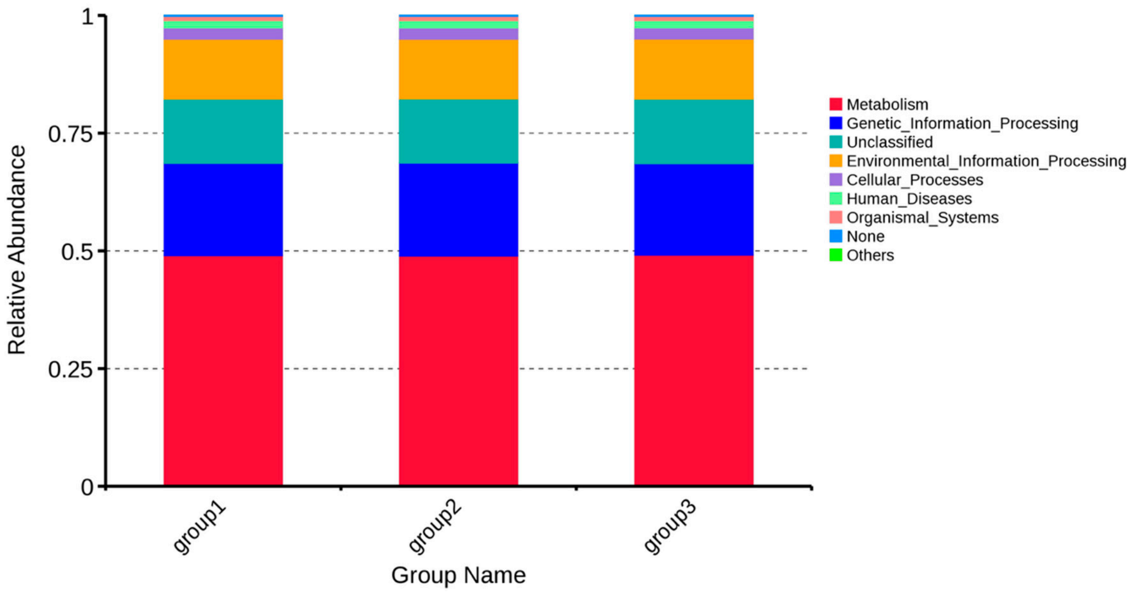

Supplementary Figure S11: Predicted functional profiles of phyllosphere bacterial communities across rice resistance groups at KEGG Level 1. Metagenome functions were predicted from 16S rRNA sequencing data using PICRUSt2. Bar graphs show the relative abundance of major functional categories (Metabolism, Genetic Information Processing, Environmental Information Processing, Cellular Processes, and Unclassified) in resistant (group 1), moderately resistant (group 2), and susceptible (group 3) rice varieties. Metabolism was the dominant category across all groups, increasing progressively from resistant (48%) to susceptible (50%) varieties, while unclassified functions were lowest in resistant varieties (10%) and highest in susceptible varieties (16%).

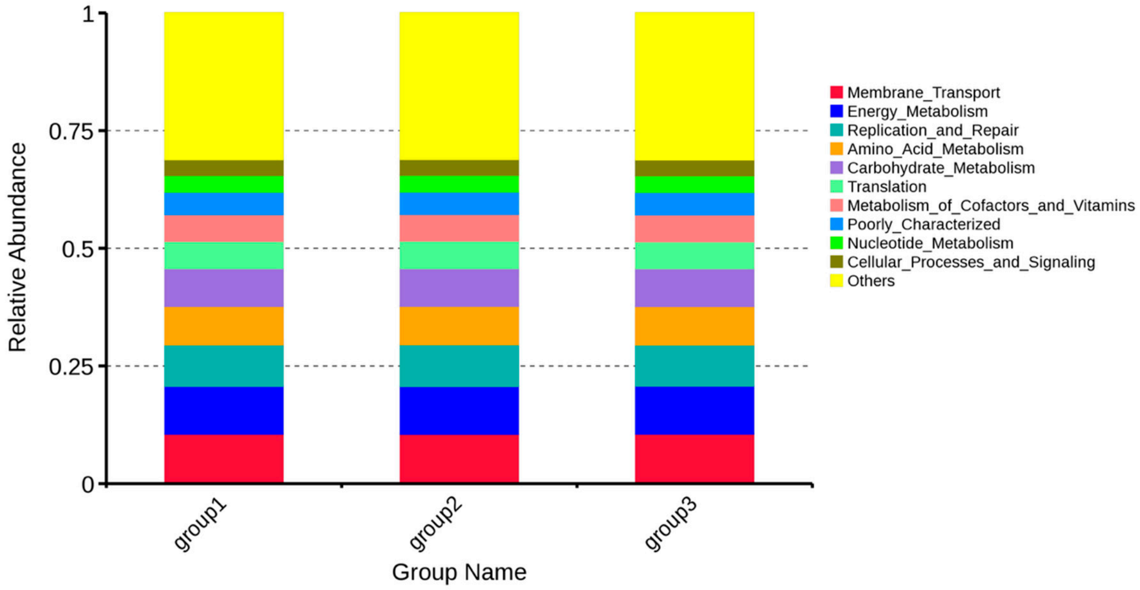

Supplementary Figure S12: Predicted functional profiles of phyllosphere bacterial communities at KEGG Level 2. Heatmap showing the relative abundance of Level 2 functional categories across resistant (group 1), moderately resistant (group 2), and susceptible (group 3) rice varieties. Translation emerged as the most abundant category in mid-resistant varieties (25%), followed by Membrane Transport (15%). Metabolism of Cofactors and Vitamins showed a progressive three-fold increase from resistant (5%) to mid-tolerant (10%) to susceptible (15%) varieties. Poorly characterized functions followed a similar gradient (5% → 10% → 15%), consistent with increased unclassified functions in susceptible varieties at Level 1.

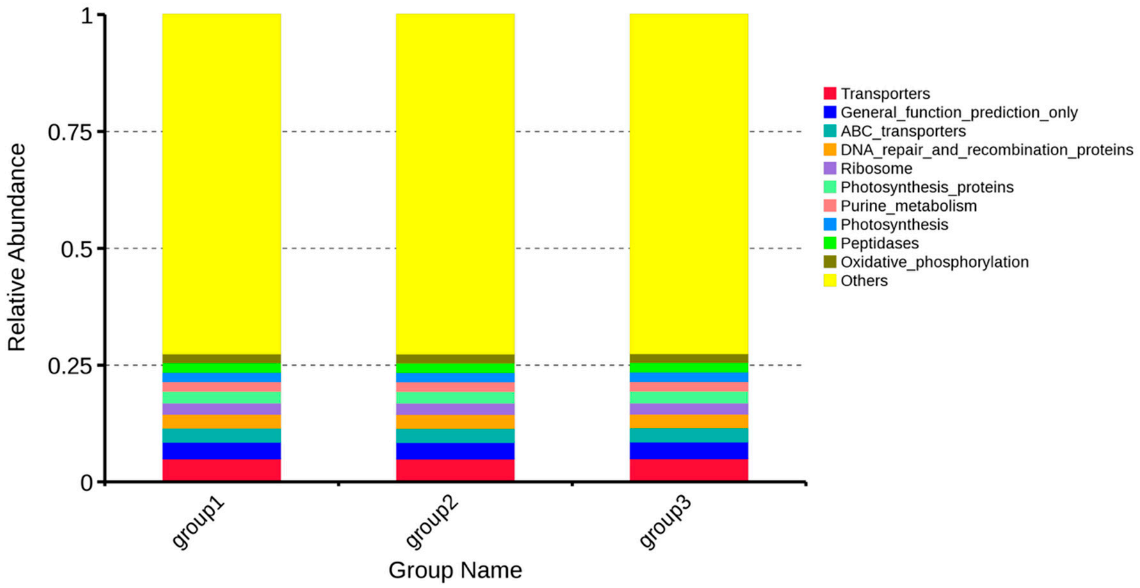

Supplementary Figure S13: Differential enrichment of specific KEGG pathways in phyllosphere bacterial communities across rice resistance groups. Bar plots showing the relative abundance of selected KEGG pathways in resistant (group 1), moderately resistant (group 2), and susceptible (group 3) rice varieties. DNA repair and recombination proteins were notably enriched in mid-tolerant varieties (>10%) compared to resistant (5%) and susceptible (5%) varieties. Photosynthesis-related pathways exhibited elevated levels in susceptible varieties, consistent with increased Cyanobacteria abundance (Supplementary Figure S1). Purine metabolism was slightly higher in resistant varieties (5%) compared to mid-tolerant and susceptible varieties (<10%).
